# Unveiling gene regulatory networks during cellular state transitions without linkage across time points

**DOI:** 10.1038/s41598-024-62850-1

**Published:** 2024-05-29

**Authors:** Ruosi Wan, Yuhao Zhang, Yongli Peng, Feng Tian, Ge Gao, Fuchou Tang, Jinzhu Jia, Hao Ge

**Affiliations:** 1 Beijing International Center for Mathematical Research, Peking University, Beijing, China; 2 Biomedical Pioneering Innovation Center, Peking University, Beijing, China; 3 School of Public Health and Center for Statistical Science, Peking University, Beijing, China; 4 Beijing Advanced Innovation Center for Genomics, Peking University, Beijing, China

**Keywords:** Computational biology and bioinformatics, Network topology, Statistical methods

## Abstract

Time-stamped cross-sectional data, which lack linkage across time points, are commonly generated in single-cell transcriptional profiling. Many previous methods for inferring gene regulatory networks (GRNs) driving cell-state transitions relied on constructing single-cell temporal ordering. Introducing COSLIR (COvariance restricted Sparse LInear Regression), we presented a direct approach to reconstructing GRNs that govern cell-state transitions, utilizing only the first and second moments of samples between two consecutive time points. Simulations validated COSLIR’s perfect accuracy in the oracle case and demonstrated its robust performance in real-world scenarios. When applied to single-cell RT-PCR and RNAseq datasets in developmental biology, COSLIR competed favorably with existing methods. Notably, its running time remained nearly independent of the number of cells. Therefore, COSLIR emerges as a promising addition to GRN reconstruction methods under cell-state transitions, bypassing the single-cell temporal ordering to enhance accuracy and efficiency in single-cell transcriptional profiling.

## Introduction

**Figure 1:**
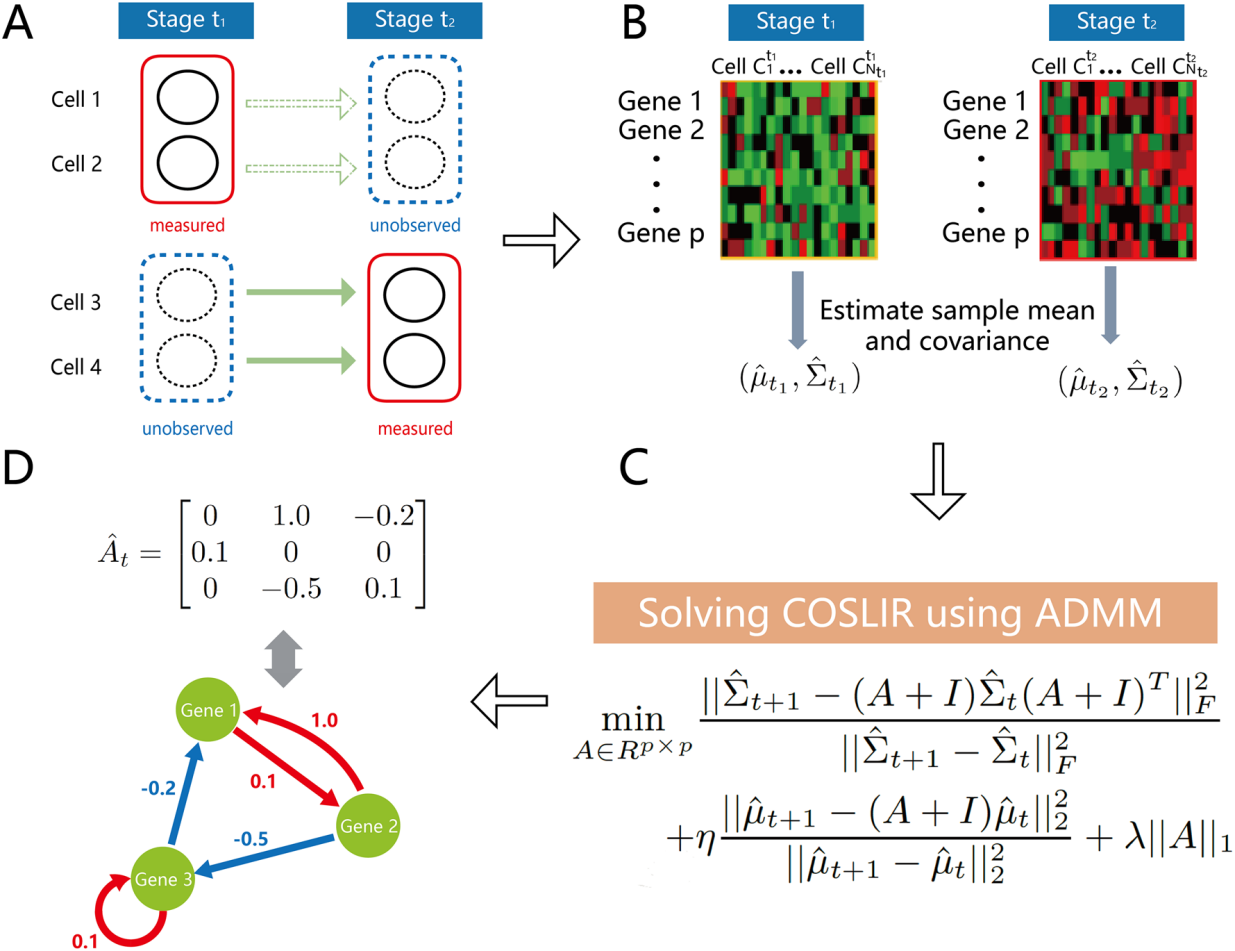
Overview of the COSLIR method for reconstructing Gene Regulatory Networks (GRNs) using time-stamped cross-sectional single-cell expression data. (**A**) Depiction of time-stamped cross-sectional data generated in single-cell experiments. Only data within the solid red rectangles were measured. (**B**) Estimation of sample mean and covariance matrix. (**C**) Solving the optimization problem in COSLIR using ADMM. (**D**) Inferred gene regulatory network among the genes represented by the estimator $\hat{A}$.

Over the past decade, the development of single-cell omics measurements has been a major breakthrough in experimental biotechnology. This innovation has generated an immense amount of data, providing critical insights into biological systems and complex diseases^1–6^. However, the sacrifice of individual cells in each assay is an inherent trade-off in single-cell omics experiments. As a result, cells measured at different times or developmental stages are from independent batches, precluding the acquisition of true longitudinal time series data. As shown in Fig. 1A, the current paradigm is limited to the generation of time-stamped cross-sectional data (TSCS) rather than comprehensive longitudinal data sets with real-time information^7^.

Despite extensive efforts over the past two decades, including the application of classical autoregressive models, differential equations, dynamic Bayesian networks, and various other dynamical models to fit both bulk and single-cell gene expression data^8–10^, challenges remain. Experimentalists have sometimes employed techniques such as cell-state synchronization or repeated sampling from larger cell cultures to generate data where trends in mean expression over time can be reasonably viewed as a time series^8,11,12^.

Even with bulk gene expression data, several computational temporal-ordering methods have been proposed^13–15^. And with the increasing of single-cell transcriptomic data, the field of reconstructing temporal information is rapidly advancing^10,16^. However, recent findings highlighted the sensitivity of gene regulatory network (GRN) inference methods to the precision of pseudo-time series construction, compromising their stability^17^. Several alternative approaches that do not rely on single-cell temporal ordering have been proposed to infer GRNs from time-stamped cross-sectional (TSCS) single-cell expression data^18–22^. However, most of these methods were limited to a single cell stage or time point, and the inferred network primarily represented the mechanism that maintains the current cell stage rather than the mechanism that drives the cell state transition along the cell lineage.

Furthermore, differential expression gene (DEG) analysis typically captures a plethora of potential upstream regulatory genes, resulting in a densely populated gene regulatory network (GRN) responsible for maintaining a single cell state, reflecting the inherent biological complexity. Conversely, GRNs that drive cell state transitions are considered sparse^23^. By identifying this smaller subset of potential upstream regulatory genes and discerning the possible regulatory relationships between them, subsequent biological functional analysis becomes more straightforward.

Hence, this paper took a direct modeling approach to characterize the Gene Regulatory Network (GRN) governing the temporal evolution of gene expression. We introduced a novel method, Covariance Restricted Sparse Linear Regression (COSLIR), which relies exclusively on the first and second moments of the measured sample (Fig. 1). Linear regression models as well as the assumption of sparsity are widely used in statistical modeling of time-series data, spanning fields such as economics, Kalman filtering, and systems biology^24–36^. The COSLIR model also employed the linear regression structure, but only used the estimated first two moments of single-cell samples at two consecutive time points.

COSLIR yielded a directed GRN, providing both signs and weights for each gene-gene interaction (Fig. 1D). The optimization problem was addressed using the alternative direction method of multipliers algorithm (ADMM^37^) (Fig. 1C). To refine the precision and stability of the estimator, bootstrapping^38^ and clipping thresholding techniques were implemented to select significant gene-gene interactions. The evaluation of COSLIR utilized published single-cell RT-PCR and RNA-seq gene expression datasets. Remarkably, COSLIR demonstrated performance on par with the best previous methods but with fewer assumptions and requirements. Furthermore, COSLIR’s running time remained nearly independent of the number of cells, ensuring its adaptability to large, recently generated datasets.

## Methods

### Models and assumptions

Let $X_t$ and $X_{t+1}$ denote two *p*-dimensional vectors representing the expression values of *p* genes within the same single cell at stages *t* and $t+1$, respectively. To model the dynamic evolution from $X_t$ to $X_{t+1}$, we introduced a $p \times p$ matrix $A_t$, where the element $(A_t)_{ij}$ in the *i*-th row and *j*-th column represents the regulatory strength from gene *j* to gene *i*.

For simplicity, we used the following linear regression model, where the dependent variable is the difference between $X_{t+1}$ and $X_t$, and the explanatory variable is $X_t$:

1 $$\begin{aligned} X_{t+1} - X_{t} = A_t X_t + \varepsilon _t, \end{aligned}$$

where the noise term $\varepsilon _t$ is a *p*-dimensional random vector, independent of $X_t$, with mean $l_t$ and finite covariance matrix $D_t$. Here, $l_t$ represents some deterministic influence induced by the environment.

However, as pointed out in the introduction, the challenge arises from the fact that once we measure $X_t$ or $X_{t+1}$, the other becomes inaccessible. There is no correspondence between the cross-sectional single-cell data collected at stages *t* and $t+1$, rendering traditional least-squares approaches ineffective. Even with an infinite sample size, the ultimate information obtainable remains limited to the exact distributions of $X_t$ and $X_{t+1}$, without providing any temporal information.

In COSLIR, the estimation of $A_t$ relies solely on the first and second moments of $X_t$ and $X_{t+1}$, and the samples are not constrained to follow a normal distribution. Let $\mu _t$ and $\Sigma _t$ denote the mean and covariance of the *p*-dimensional random vector $X_t$. The following equations can be derived from (1):

2 $$\begin{aligned} \Sigma _{t+1}= & {} (A_t+I)\Sigma _t (A_t+I)^T + D_t ;\nonumber \\ \mu _{t+1}= & {} (A_t+I)\mu _t+l_t , \end{aligned}$$

where *I* is the identity matrix.

Although the general methods of moments to infer parameters have been widely used in statistics, one always needs the covariance between the predictor and response when estimating parameters in linear regression model, which is absent in time-stamped cross-sectional data. Therefore, we only had equation (2) , which is underdetermined with respect to $A_t$, implying infinitely many solutions for $A_t$ even when $\Sigma _{\cdot }$, $\mu _{\cdot }$, $l_{\cdot }$, and $D_{\cdot }$ are known. Then we assumed sparsity in $A_t$, and small values for both $l_t$ and $D_t$, indicating a dominance of linear relationships in this scenario. Sparsity is a common assumption in statistical learning^24,25,27^, aligning with observations in single-cell biology^23^. Following the principles of compressed sensing with nonlinear observations^28^ and applying the penalty method, we obtained the sparse matrix $A_t$ by solving the non-convex optimization problem outlined in (3) (Fig. 1C, Supplementary Material and Methods):

3 $$\begin{aligned} \begin{aligned} \min \limits _{A \in \mathbb {R}^{p \times p}} \frac{||\hat{\Sigma }_{t+1} - (A+I)\hat{\Sigma }_t(A+I)^T||_F^2}{||\hat{\Sigma }_{t+1}-\hat{\Sigma }_t||_F^2} + \eta \frac{||\hat{\mu }_{t+1} -(A+I)\hat{\mu }_t||_2^2}{||\hat{\mu }_{t+1}-\hat{\mu }_t||_2^2} + \lambda ||A||_1, \end{aligned} \end{aligned}$$

where $\hat{\Sigma }_{\cdot }$ and $\hat{\mu }_{\cdot }$ are the estimates of the covariance and mean of $X$ (Fig. 1B). $||\cdot ||_F$ denotes the Frobenius norm, and $||\cdot ||_1$ denotes the sum of the absolute values of all elements in a matrix.

In practical scenarios, we utilized the estimators $\hat{\mu }$ and $\hat{\Sigma }$ when the exact mean and covariance are unknown. For relatively large sample sizes, the naive sample mean and covariance estimators are recommended. However, in cases where the sample size is small compared to the number of components, resulting in potentially high variance, the sample mean and covariance may yield inaccurate estimators for $A_t$. In such situations, the use of high-dimensional techniques^39–42^ was recommended to obtain more accurate estimates of mean and covariance. In the context of single-cell RNA-seq data, preliminary imputation techniques can be used to remove technical noise, such as dropout or batch effects^43^, before estimating the mean and covariance.

For simplicity, in the subsequent numerical experiments, we exclusively utilized the sample estimators $\hat{\mu }$ and $\hat{\Sigma }$. However, a correction was applied to the covariance matrix using

4 $$\begin{aligned} \tilde{\Sigma } = (1-\alpha )\hat{\Sigma } + \alpha I, \end{aligned}$$

where *I* is an identity matrix, and $\alpha =0.01$ ensures positive definiteness.

Two tuning parameters, $\eta$ and $\lambda$, play crucial roles. The first two terms in (3) regulate the quantitative relationships between the mean and covariance of $X_t$ and $X_{t+1}$ based on (1), while $\lambda$ controls the sparsity of $A_t$.

It is important to note that COSLIR should be applied to each pair of consecutive cell stages or time points and does not presuppose the gene regulatory network to be invariant with respect to time *t*.

### Solving the optimization problem using ADMM

We employed an efficient numerical algorithm, the Alternating Direction Method of Multipliers (ADMM^37^), to solve (3). Initially, we introduced two constraints, $A+I=(B+C)/2$ and $B=C$, and subsequently, we partitioned the variables into three components for the application of ADMM. Introducing *B* and *C* is a common technique that significantly simplifies each substep in the ADMM algorithm.

The optimization problem can then be reformulated as follows:

$$\begin{aligned} \begin{aligned} \text {min}_{A,B,C}& \mathscr{L}(A, B, C, \Pi _1, \Pi _2)\\ \text {s.t.}\qquad&A+I=\frac{B+C}{2}, B=C, \text {where} \\ \mathscr {L}(A, B, C, \Pi _1, \Pi _2) =&\frac{||\hat{\Sigma }_{t+1} - B\hat{\Sigma }_t C^T||_F^2}{||\hat{\Sigma }_{t+1} - \hat{\Sigma }_t||_F^2} + \eta \frac{||\hat{\mu }_{t+1} -\frac{B+C}{2}\hat{\mu }_t||_2^2}{||\hat{\mu }_{t+1} -\hat{\mu }_t||_2^2} \\&\quad + \lambda ||A||_1 + \langle B-C, \Pi _1 \rangle + \frac{\rho }{2}||B-C||_F^2 \\&\quad + \langle A + I - \frac{B+C}{2}, \Pi _2 \rangle + \frac{\rho }{2}||A+I - \frac{B+C}{2}||_F^2. \end{aligned} \end{aligned}$$

In each iteration using ADMM, we simply minimised and updated *A*, *B*, *C* separately. Such an iteration will lead to a reasonably converged state (with an adaptively tuned step size). More explicitly, the algorithm is written in Algorithm (1).

**Algorithm 1:**
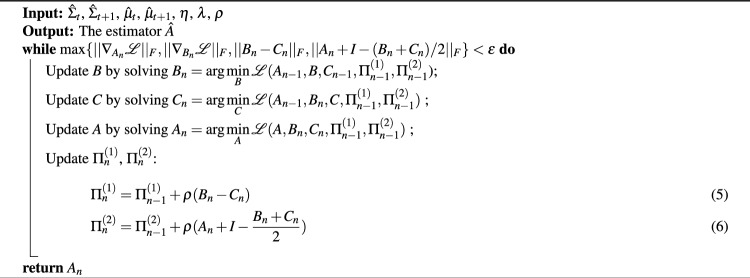
ADMM for COSLIR

Importantly, for the subproblems involving the update of *A*, *B*, *C*, the objective function is at most quadratic (and hence convex) when focusing on a single variable. This characteristic implies that, under the optimality condition for each subproblem, we only need to solve linear equations and can consistently obtain closed-form solutions. This property significantly contributes to the efficiency of our algorithm.

Furthermore, we have incorporated optimization techniques into our published code to accelerate the convergence rate of ADMM. These techniques, proven to be effective in both simulation studies and real data experiments^44,45^, enhance the performance of the algorithm.

Upon solving the optimization problem, the non-zero elements in $A_t$ denote the regulatory relationships between genes (Fig. 1D). It is noteworthy that (3) constitutes a non-convex optimization problem, potentially leading to multiple local optima. However, we observed that initiating the ADMM algorithm with a zero matrix consistently yields a reasonable solution.

### Bootstrapping and clipping thresholding

Estimating the mean and covariance introduces potential imprecision in predicting $A_t$. To enhance both robustness and precision-of greater interest to experimentalists^17^-we employed the nonparametric bootstrapping technique^46^. In essence, we repeated the following procedure multiple times and aggregate all assembled estimators for $A_t$ into an ensemble: initially, perform random sampling with replacement from the two collections of samples at different stages or times, forming two new collections of observations; subsequently, apply COSLIR to the new sample collections to acquire a fresh estimator for $A_t$. Finally, only those non-zero elements with a confidence level (repetition ratio with the same sign) surpassing a specified threshold were retained in the ultimate estimator of the interaction matrix $A_t$.

Furthermore, in practical scenarios, we implemented an additional clipping thresholding procedure on the output to discard non-zero entries with very small absolute values below a certain threshold (entries with negligible values in $\hat{A}_t$ are likely due to noise). Following clipping thresholding and bootstrapping, we regarded the remaining non-zero entries as statistically significant, with their mean constituting the final estimator.

### Model selection and evaluation

There are four hyperparameters to determine in the aforementioned process: two ($\lambda$ and $\eta$) in the optimization problem and two (confidence threshold and clipping threshold) in the bootstrapping process.

$\lambda$ controls the sparsity of $A_t$, while $\eta$ governs the noise term in (3). As our model is unsupervised due to the absence of simultaneously measured $X_t$ and $X_{t+1}$, cross-validation is impractical. To address this challenge, we empirically relied on three indices as criteria for model selection:

7 $$\begin{aligned} e_{\Sigma }(\hat{A})&= ||\hat{\Sigma }_{t+1} - (\hat{A_t}+I)\hat{\Sigma }_t(\hat{A}+I)^T||_F / ||\hat{\Sigma }_{t+1} - \hat{\Sigma }_t||_F \\ e_{\mu }(\hat{A})&= ||\hat{\mu }_{t+1} - (\hat{A_t}+I)\hat{\mu }_t||_2 / ||\hat{\mu }_{t+1} - \hat{\mu }_t||_2 \\ s_0(\hat{A})&= \frac{\#\,\{{\text{non-zero elements of}} \,\hat{A}\}}{p^2}. \end{aligned}$$

Here, $e_{\Sigma }$ and $e_{\mu }$ quantify the small noise terms in (2), while $s_0(\hat{A})$ gauges the sparsity of $\hat{A_t}$. We selected the model with all three indices minimized, indicating the sparsest matrix $A_t$ with $e_{\Sigma }$ and $e_{\mu }$ below a reasonable level. Our simulation study demonstrated the effectiveness of this proposed criterion (see Supplementary Results, Supplementary Fig. S2, S3, Supplementary Tables S2, S3). To simplify the process, during bootstrapping, we initially determined the hyperparameters $\lambda$ and $\eta$ using the entire sample and then used only these determined values for subsequent analyses. Throughout the paper, we typically set $\lambda = 10^{-6}$ and $\eta =5$.

In the oracle case of the simulation study, a substantial gap always exists between the values of the non-zero elements, making the determination of the clipping threshold straightforward. However, in the sample case, it is less clear which value of the clipping threshold to choose. We proposed determining the clipping threshold based on the trade-off between the three indices of the criterion in (7). Specifically, we let $\hat{A_t}(\varepsilon )$ be the adjusted estimator after taking $\varepsilon$ as the clipping threshold. We determined the threshold $\varepsilon$ by choosing the sparsest $\hat{A_t}(\varepsilon )$ with $e_{\Sigma }(\hat{A_t}(\varepsilon )) + \eta e_{\mu }(\hat{A_t}(\varepsilon ))$ below a reasonable level. Similar to the $\lambda$ and $\eta$ hyperparameters, this threshold was determined before bootstrapping on the full sample.

For the confidence threshold, it can be tuned, but we additionally required it to be greater than 0.5 for it to make sense.

In the simulation studies, we consider the non-zero elements with the correct sign as *correctly recovered*. To evaluate performance, we adopted the commonly used criteria precision and recall, i.e.,

8 $$\begin{aligned} \text {precision}= & {} \frac{\#\{\text {correctly recovered non-zero elements}\}}{\#\{\text {non-zero elements in}\hat{A_t}\}};\,\, \end{aligned}$$

9 $$\begin{aligned} \text {recall}= & {} \frac{\#\{\text {non-zero elements recovered correctly}\}}{\#\{\text {non-zero elements in true}\,A_t\}}\,\, . \end{aligned}$$

In real datasets, when comparing COSLIR with other methods, instead of using the previous precision and recall, we used a slightly modified Early Precision from BEELINE^17^ for evaluation. Early precision is the number of true positives in the most significant *k* edges (which is also the precision of this top-k network).

### Simulation settings

In the simulation studies, we first generated the mean and covariance of $X_t$, the ground-truth interacting matrix *A*, as well as the mean and covariance of the noise term. Subsequently, using (2), we calculated the mean and covariance of $X_{t+1}$. The sample data was then generated from the normal distribution based solely on the mean and covariance of $X_t$ and $X_{t+1}$. Furthermore, in the oracle case, we only input the mean and covariance at the two stages into our algorithm. In contrast, in the sample case, we provided the algorithm with different samples at the two stages without any time-series correspondence.

In order to make sure the generated covariance matrix $\Sigma _t$ of $X_t$ is positive-definite, we used the formula $\Sigma _t = P \Lambda P^T$, where $\Lambda \in \mathbb {R}^{p \times p}$ is a diagonal matrix with $\Lambda _{ii} = \exp {\left( \frac{i}{p}\right) }$, $i=1,2,\cdots ,p$, and $P = I + R$, where *I* is the identity matrix. For each round of simulation, the elements of *R* were sampled independently from standard normal distributions. The true interacting matrix $A_t \in \mathbb {R}^{p\times p}$ is a sparse matrix, where only $10\%$ of the elements were non-zero and randomly generated from $\mathcal {N}(0, 1)$ independently. The elements of the mean $\mu _t \in \mathbb {R}^p$ of $X_t$ were independently generated from $\mathcal {N}(0, 100)$. The mean of each element of the noise term $\varepsilon _t \in \mathbb {R}^p$ was set to 0.1, and the covariance matrix $D_t$ of $\varepsilon _t$ was set to be a diagonal matrix with $(D_t)_{ii}=0.01$ for each *i*. Subsequently, $\mu _{t+1}$ and $\Sigma _{t+1}$ were calculated using (2).

The numerical experiments were repeated 100 times in the oracle case and 50 times in the sample case. The bootstrapping procedure was repeated 50 times in each sample experiment. The obtained values of $\eta$, $\lambda$, and the clipping threshold were summarized in Supplementary Table S1.

### Real datasets

The RT-PCR dataset^47^ included 442 single cells selectively collected from early mouse embryonic development. The dataset comprised mRNA expression levels of 48 genes, including 27 transcription factors, 19 known marker genes, and 2 housekeeping genes for normalization.

We used two experimental single-cell RNA sequencing (scRNA-seq) datasets: one involving human embryonic stem cell (hESC) differentiation to definitive endoderm^48^, and the other focused on mouse embryonic stem cell (mESC) differentiation to primitive endoderm^49^. Genes within the RNA-seq datasets were selected following the same strategy employed in BEELINE^17^. For the mESC datasets, we identified 970 genes, respectively. The datasets comprised 90 (0h), 68 (12h), 90 (24h), 82 (48h), and 91 (72h) cells across 5 time points. In the case of hESC datasets, with 814 genes, respectively, the dataset consisted of 92 (0h), 102 (12h), 66 (24h), 172 (36h), 138 (72h), and 188 (96h) cells spanning 6 time points. It’s essential to note that data from BEELINE were already normalized; thus, we utilized it post gene selection. The COSLIR bootstrapping procedure was repeated 50 times.

The single-cell human blood atlas dataset was from the official Peripheral Blood Mononuclear Cell Multiome dataset^50^. Wo focused on the three lineages of HSPC cells: HSPC$\rightarrow$B, HSPC$\rightarrow$Erythrocyte and HSPC$\rightarrow$Monocyte. There were 6044 cells in total, with 1909 HSPC cells, 1448 B cells, 1747 Erythrocyte cells and 940 Monocyte cells. For gene selection, we first selected top 2000 high variable genes. Then for each developmental lineage we determined DEGs using Seurat with default parameter. Finally we selected 623 genes during HSPC$\rightarrow$B, 893 genes in HSPC$\rightarrow$Erythrocyte and 949 genes in HSPC$\rightarrow$Monocyte. We took the union of them, 1475 genes in total.

We did not have any human or animal involved in this study.

### Re-scaled values for selecting the most influential regulatory interactions

We recommended an additional processing step for real data analysis using COSLIR. In the analysis of single-cell expression data, one might be particularly interested in interactions that contribute more to changes in mean expression, i.e., differentially expressed genes (DEGs). Therefore, we introduced a rescaled value

$$\begin{aligned} (\tilde{A})_{ij} = \frac{(|\hat{A}|)_{ij}|\hat{\mu }_1|_j}{|(\hat{\mu }_1)_i - (\hat{\mu }_2)_i|}, i,j=1,2,...p, \end{aligned}$$

to select the most influential regulatory gene-gene interactions. This technique was applied solely to the final estimator obtained after the bootstrapping procedure.

## Results

### Simulation study

In this study, we evaluated the performance of COSLIR through simulation studies, examining both the oracle and sample cases. The oracle case assumes knowledge of the true mean and covariance matrix, while the sample case involves working with random samples only (see simulation settings in Methods).

We did not intend to evaluate COSLIR on simulated single-cell expression data. What we really want to validate here is the optimization algorithm (3). Even when the data is generated by the linear model (1), there is no theoretical guarantee that the solution of the optimization algorithm (3) recovers the ground-truth matrix *A* in the absence of the cross-talk between the data at time *t* and time $t+1$.

#### Simulation results

In the oracle cases, we evaluated three indices proposed in Eq. (7) across different values of $\lambda$ and $\eta$, including $e_{\Sigma }(\hat{A})$ for the error of estimated covariance matrix, $e_{\mu }(\hat{A})$ for the error of estimated mean, and $s_0(\hat{A})$ for sparsity. The results exhibited robustness to variations in $\eta$, and our criteria effectively guided the determination of optimal or suboptimal values for $\lambda$ (see Supplementary Figures S1, S3, Table S1, S3).

**Figure 2:**
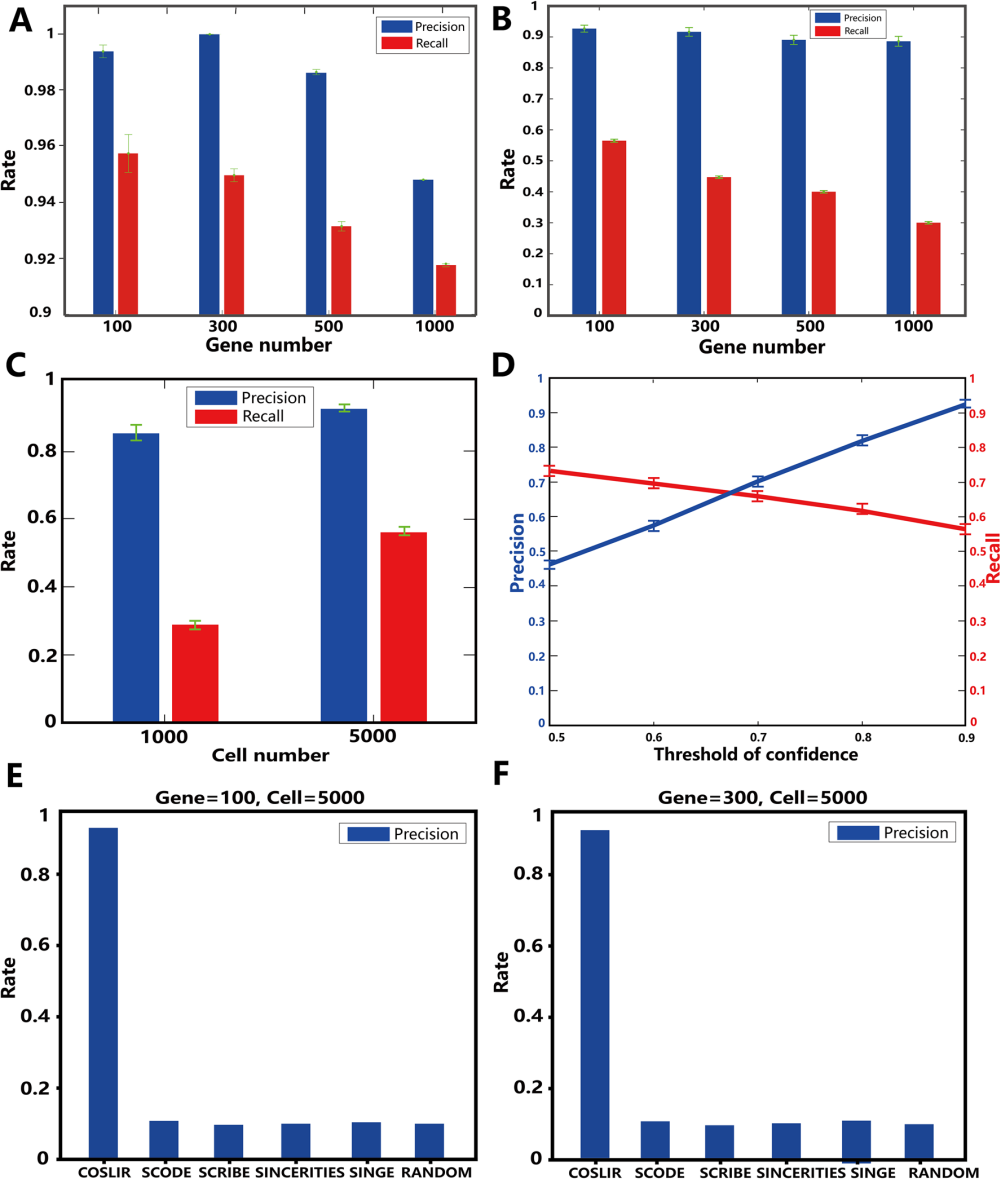
The performance of COSLIR is rigorously evaluated through a simulation study that includes both oracle cases (**A**) with known true mean and covariance matrix, and sample cases (**B**–**D**) where only random samples are available. In the oracle case, precision and recall were shown (**A**). In the sample case, precision and recall vary with the number of genes (**B**), the number of cells (**C**) and the confidence threshold (**D**). The number of cells in (**B**, **D**) is 5000. The number of genes in (**C**, **D**) is 100. The precision of COSLIR is compared to four existing algorithms and random guess (**E**, **F**). The confidence threshold is set to 0.9 in (**B**, **C**, **E**, **F**).The clipping threshold is always 0.01. More detailed results can be found in Supplementary Figs. S2–S4 and Tables S1–S5.

Fig. 2A vividly demonstrated the almost exact recovery of the estimator obtained by COSLIR in oracle cases. This was true even when the data dimension reaches 500 and the number of gene-gene interactions in $A_t$ to be inferred increases to 2.5 $\times$ $10^5$. Notably, the precision, recall and exact values of the estimator closely mirrored the ground truth values (see Supplementary Table S2). This robust performance provided confidence in the applicability of the method to simulated sample data, where separate estimation of $\Sigma _t$, $\Sigma _{t+1}$, $\mu _t$ and $\mu _{t+1}$ is mandatory.

Fig. 2B-D illustrated how COSLIR’s performance varied with the number of genes, cells and confidence threshold in the sample cases. Even with a large number of genes, high precision can be achieved by setting a sufficiently high bootstrapping confidence threshold (Fig. 2B,D). Although this comes at the cost of a lower recall compared to the scenario with a smaller number of genes (already lower than in the oracle case), as shown in Fig. 2B, it’s crucial to recognise that the matrix $A_t$ contains an exceptionally high number of gene-gene interactions (square of the dimension). Despite the reduced recall, the actual number of successfully recovered gene interactions remained substantial.

Sample size significantly influenced COSLIR’s performance, with increasing precision and recall as sample size grows (Fig. 2C). The precision-recall trade-off when tuning the bootstrapping confidence threshold (Fig. 2D, Supplementary Fig. S4) can be tailored to specific needs in real applications. Generally, a higher confidence threshold is recommended for precision-focused studies, typical in most experimental science studies. In addition, the sparser the true matrix $A_t$, the better the performance, as fewer samples are required (Supplementary Fig. S3).

We compared COSLIR with four established regression-based algorithms for Gene Regulatory Network (GRN) reconstruction: SINCERITIES, SCODE, SCRIBE, and SINGE, as summarized in BEELINE^17^. The precision and AUC of precision-recall curve of COSLIR was much higher than those of the four existing algorithms (Fig.2E, F, Supplementary Figure S5). It was not surprising, since the simulation data was generated based on Eq. 1. Although the four other algorithms are all based on similar regression models, the detailed assumptions and expressions are not the same.

### Results on real datasets during early embryo development

Revealing gene expression patterns in early embryos is crucial for understanding cellular developmental processes^47,51–54^. However, due to the limitations of real-time experimental measurements, the detailed regulatory mechanisms that control this nascent stage of life remain unknown. In this study, we used COSLIR to infer the gene regulatory networks that govern cell fate decisions.

#### Single-cell RT-PCR dataset

**Figure 3:**
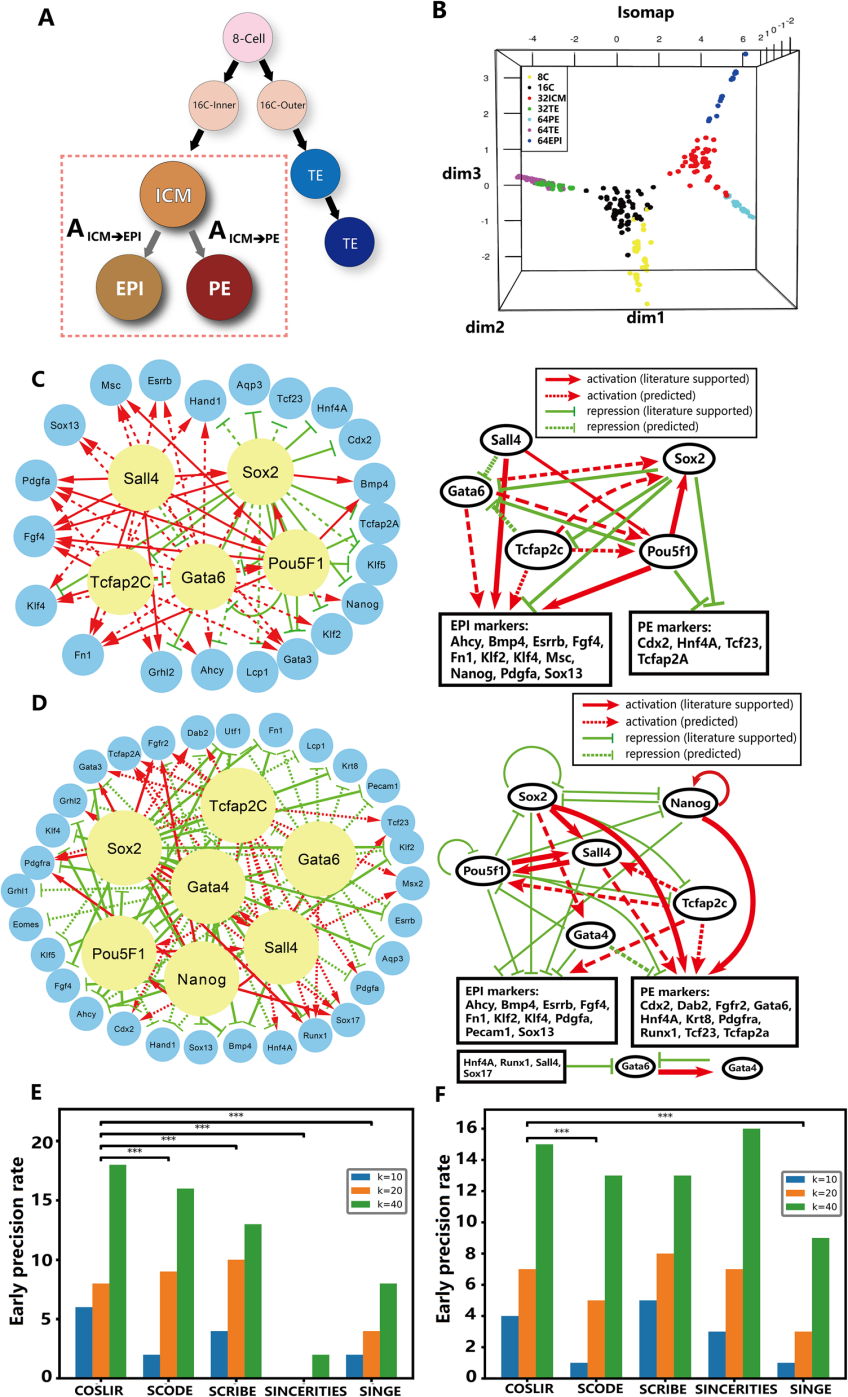
Overview of the analysis of real single-cell RT-PCR expression data using COSLIR. (**A**) Developmental lineage tree representing early mouse embryonic development. (**B**) Data visualization using Isomap. (**C**) Inferred gene regulatory networks (GRNs) directing ICM cells towards the EPI fate, accompanied by a corresponding sketch map. (**D**) Inferred GRNs directing ICM cells to the fate of PE, accompanied by its sketch map. (**E**, **F**) Early precision comparison of COSLIR with four existing algorithms during the two cell lineages: ICM cells towards the EPI fate (**E**) and the PE fate (**F**). Here, the clipping threshold is 0.01, the confidence threshold is 0.8, and the threshold of the rescaled values we chose is 0.25 for ICM to EPI and 0.1 for ICM to PE. More details can be found in Supplementary Figures S6–S7 and Table S5.

We began our analysis by examining a dataset of published single-cell gene expression data during mouse embryonic development, obtained using the RT-PCR technique^47^. This dataset included 442 single cells selectively collected from early mouse embryonic development, spanning 7 developmental stages: Zygote, 2-cell stage, 4-cell stage, 8-cell stage, 16-cell stage, morula stage, and blastocyst stage. Of particular note is the blastocyst stage, where embryos manifest three distinct cell types-trophectoderm (TE), primitive endoderm (PE), and epiblast (EPI)-each characterized by unique gene markers and expression patterns^55^. In our analysis of the data from the 8-cell stage to the blastocyst stage (Fig. 3A and B, Supplementary Table S5), we used the non-linear dimension reduction method ISOmap^56^ instead of the linear dimension reduction method PCA used in Guo et al.^47^. This approach revealed two crucial cell fate decisions in the dataset: (1) the segregation of inner and outer cells at the 16-cell stage, leading to the formation of inner cell mass (*ICM*) and trophectoderm (*TE*), and (2) the subsequent differentiation of the *ICM* into primitive endoderm and epiblast.

The dataset comprised mRNA expression levels of 48 genes, including 27 transcription factors, 19 known marker genes, and 2 housekeeping genes for normalization, during the first two cell fate decisions of the early mouse embryo. Our analysis focused on the data of 46 non-housekeeping genes, and the raw data were rescaled through log transformation. Due to the prevailing dominance of maternal gene degradation in gene expression variation during the initial cell fate decision, in contravention of COSLIR’s requirement, our analysis using COSLIR was limited to the second cell fate decision.

Fig. 3C,D provided an overview of the two inferred gene regulatory networks (GRNs) using Cytoscape^57^. Using COSLIR, we inferred a set of directed gene regulatory relationships corresponding to the positive or negative elements in the $A_t$ matrices, indicating activated or inhibited regulatory links between genes. The identified upstream regulatory genes and major regulatory relationships were consistent with existing knowledge about the second cell fate decision during early embryonic development^51,58–60^. Key upstream regulators such as *Sall*4, *Sox*2, *Pou*5*f*1, *Gata*6, and *Tcfap*2*c* played critical roles in the inferred GRN, driving *ICM* cells toward the *EPI* fate, activating *EPI* markers and inhibiting *PE* markers (Fig. 3C). Similarly, in the inferred network that directs *ICM* cells toward the *PE* fate, key upstream regulators such as *Sall*4, *Sox*2, *Pou*5*f*1, *Gata*4, *Tcfap*2*c*, and *Nanog* acted to inhibit *EPI* and activate *PE* markers (Fig. 3D). All identified upstream regulatory genes and their target markers were well established for their roles in early embryonic development^47,61^. In addition, through literature and database searches (ChIP-atlas(ESC), BioGRID, and TRRUST), we found that nearly half of the inferred regulatory relationships were reported experimentally (Supplementary Figures S6, S7), with many validated by multiple databases.

Early precision serves as the metric for evaluating these algorithms, quantified as the count of correctly inferred gene regulatory relationships among the top *k* relationships with the highest weights. For COSLIR, we utilized rescaled values, indicative of the significance of each inferred gene regulatory relationship for the differential expression of the target gene (refer to Methods for detailed information). Fig. 3E-F compared COSLIR with the four existing algorithms, using early precision. It illustrates that COSLIR competed favorably with existing methods using pseudo-time construction. The p-values were calculated by Wilcoxon signed rank test, using all the early precision at each $k=1,2,\cdots ,40$.

The inferred regulatory relationships not cataloged in the three databases may still be accurate predictions (Supplementary Figure S7). For example, the regulatory link from *Sox*17 to *Gata*6 reported by Niakan et al.^62^ was not included in the three databases, but was successfully predicted by COSLIR. It’s important to note that we used only the *ICM* and *EPI* or *ICM* and *PE* datasets to infer the gene regulatory networks independently. For example, despite the modest fold change of *Gata*6 from the *ICM* stage to the *PE* stage (only 1.03), it was recognized as a crucial marker gene for the *PE* stage compared to its expression in the *EPI* stage. Remarkably, COSLIR accurately inferred this relationship without reference to gene expression data from the *EPI* stage, underscoring the robust and independent predictive power of COSLIR.

#### Single-cell RNA-seq datasets

We conducted a comprehensive comparison of COSLIR with four established regression-based algorithms for Gene Regulatory Network (GRN) reconstruction: SINCERITIES, SCODE, SCRIBE, and SINGE, as summarized in BEELINE^17^. Our analysis encompassed two experimental single-cell RNA sequencing (scRNA-seq) datasets: one involving human embryonic stem cell (hESC) differentiation to definitive endoderm^48^, and the other focused on mouse embryonic stem cell (mESC) differentiation to primitive endoderm^49^. Given the multiple time points within these datasets, we reconstructed GRNs for each pair of consecutive time points.

**Figure 4:**
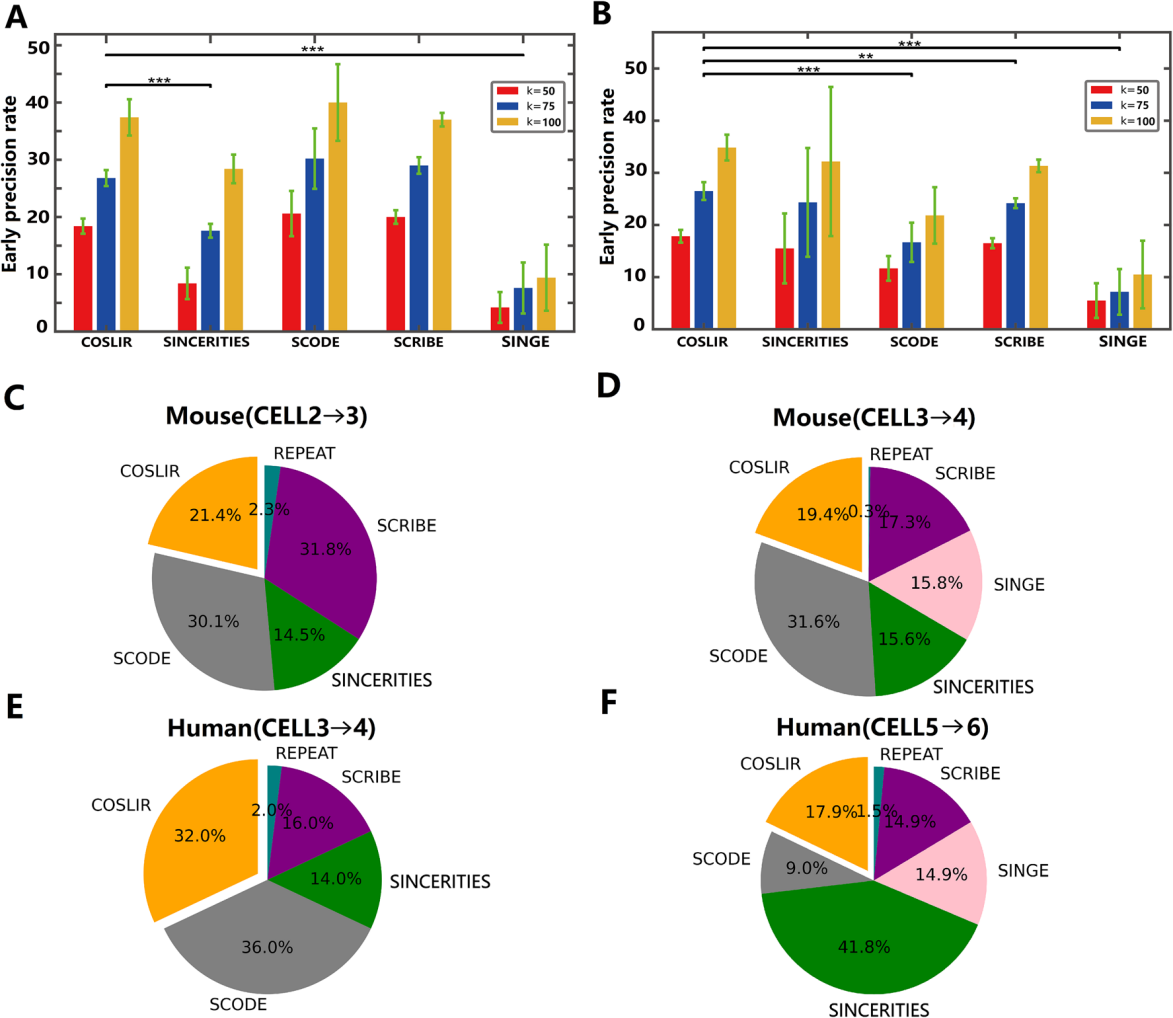
Comparative analysis of various methods on mESC and hESC single-cell RNA-seq datasets. (**A**, **B**) Display of early precision across different methods and various values of *k* in mESC (**A**) and hESC (**B**) datasets. (**C**–**F**) Pie charts illustrating the distribution of database-supported gene regulatory relationships predicted by each method among all database-validated predictions between consecutive time points during mESC and hESC development. Notably, in (**C**, **E**), the SINGE method does not predict any gene regulatory relationships supported by the database. We use the cell type-specific ground truth in BEELINE^17^.

To evaluate the reconstructed networks, we employed cell-type-specific networks^17^ as the ground truth networks (Fig. 4). For a more in-depth comparison, including functional interaction networks (STRING) and additional cell-type-specific ChIP-Seq data, refer to Supplementary Figures S8-S11.

In Fig. 4A-B, the early precision of various methods at different values of *k* was presented for both datasets, using the cell type-specific ground truth in BEELINE^17^. The mean and error bar of early precision were computed from the outputs of each algorithm for each pair of consecutive time points. Notably, it was evident from the results that COSLIR’s performance competed favorably with the four existing methods. The p-values were calculated by Wilcoxon signed rank test, using all the early precision at $k=1,\cdots ,100$ for each two consecutive time points.

Furthermore, the sets of gene-gene regulatory interactions inferred by various methods on high-dimensional single-cell RNAseq data exhibited minimal overlap with each other (Fig. 4C, D, and Supplementary Figures S12-S13). This suggested that the individual accuracy of any existing method was notably limited, and COSLIR emerged as a valuable addition to the existing toolkit of methods.

### Scalability of COSLIR via single-cell Human blood atlas

The throughput of single-cell experiments is experiencing a remarkable increase. To effectively handle the surge in data throughput, computational methods must be designed with scalability at the forefront. Fortunately, COSLIR is inherently scalable, relying solely on the estimation of the first and second moments of the samples. This inherent scalability ensures the method’s applicability at a large scale.

**Figure 5:**
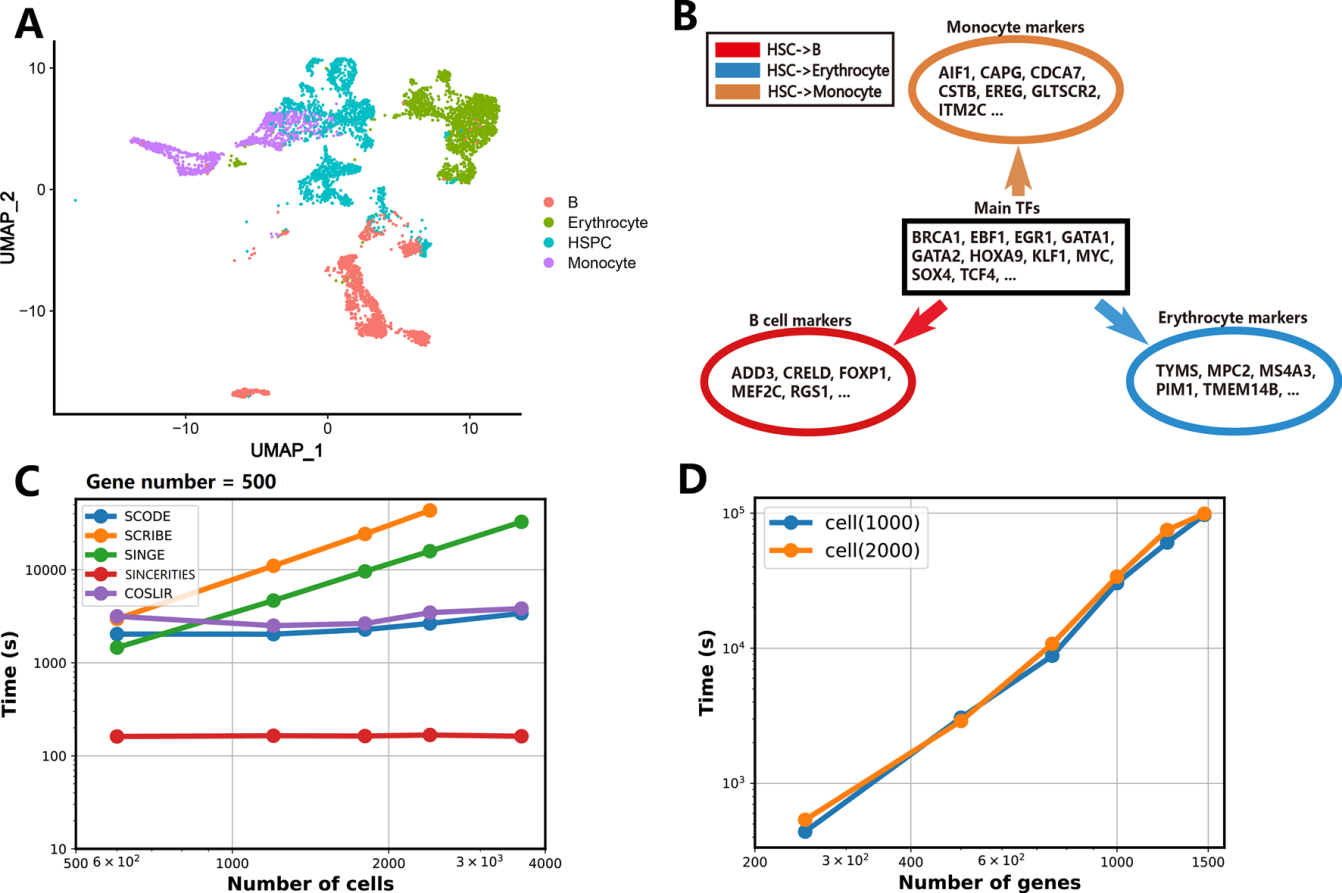
COSLIR being applied on single-cell Human blood atlas. (**A**) UMAP visualizations of human blood cell embeddings, with colors indicating distinct cell types. (**B**) COSLIR-inferred major upstream regulatory transcription factors (TFs) and downstream cell-type markers. (**C**) Time costs of different methods applied to subsampled atlases with varying cell numbers. (**D**) Time costs of COSLIR applied to subsampled atlases with different gene numbers. Further comprehensive verification with relevant literature can be found in Supplementary Tables S7–S10.

We evaluated COSLIR using the official Peripheral Blood Mononuclear Cell Multiome dataset^50^. The majority of gene-regulatory interactions, identified by COSLIR with the highest rescaled values, corresponded to significant biomarkers and pathways implicated in blood development (Fig. 5A, B, Supplementary Tables S6-S7, Supplementary Figure S14). Notably, COSLIR demonstrated near independence of computation time from the cell number (Fig. 5C), showcasing its scalability. While the computational time of COSLIR exhibited an exponential increase with the number of genes (Fig. 5D), it’s important to note that such behavior is a common trait shared by almost all methods for inferring gene regulatory relationships^17^.

## Conclusion and Discussion

In the current era, the convergence of machine learning and network biology presents both invaluable opportunities and challenges, especially in the field of gene regulatory network inference^63^. In this paper, we presented COSLIR, an optimization-based model designed for gene regulatory network inference. A distinctive feature of our approach is its minimal input requirements, relying only on the estimated mean and covariances of the samples at consecutive time points in single-cell expression data. Despite its simplicity, COSLIR produces directed gene regulatory networks with weights and signs, making it a versatile tool applicable to diverse sample distributions. In simulation studies, COSLIR demonstrated remarkable accuracy in recovering true networks under oracle conditions, and its performance remained robust in sample cases, particularly with the aid of bootstrapping to achieve near-perfect precision. In real data analyses, COSLIR successfully identified important upstream regulatory genes and gene-gene interactions in single-cell RT-PCR and RNA-seq datasets, shedding light on early mouse and human embryonic development.

However, in practical applications, several factors may affect the performance of COSLIR. Multi-scale data across genes may require normalization before applying COSLIR. While it is recommended to use a correlation matrix rather than a covariance matrix, this introduces some inherent bias. Gene selection is another challenge, as the computational time for COSLIR becomes significant when the number of genes exceeds 1000^17^. Common strategies involve the selection of highly variable genes, often in conjunction with transcription factors. Also in our formulation modeling the difference between the stage *t* and $t+1$, we assumed that the cells at stage *t* and $t+1$ are homogeneous. Hence, COSLIR should be applied after the cells being clustered into homogenous populations, just as we have done in this paper when analyzing the real single-cell data along each developmental lineage.

Another important consideration is whether the data strictly adhere to the linear model (1). While the linear regression model is the predominant choice for gene regulatory network (GRN) reconstruction from single-cell expression data^32–34,64,65^, it’s worth noting that linearity justification is inherently missing from time-stamped cross-sectional data. In statistical learning, the default inclination is to choose the linear model unless there is compelling evidence of high nonlinearity. Empirically, as long as the data don’t deviate significantly from linearity, the performance of statistical inference by the linear model remains robust^66^. In addition, the linear model offers a distinct advantage in terms of interpretability, following the principle that simplicity is often synonymous with effectiveness.

To improve accuracy, the GRN reconstruction method can be synergistically integrated with existing prior knowledge by incorporating additional databases or information^67^. Recent advances, such as RNA velocity analysis, have demonstrated the potential to recover some temporal information^68^. Therefore, our future research path is to explore the merging of COSLIR with existing database knowledge and bioinformatic methods, leveraging these complementary approaches for improved precision and insight.

### Supplementary Information

Supplementary Information.

## Data Availability

The proposed algorithm and dataset are available at https://github.com/Ge-lab-pku/COSLIR.

## References

[1] Lu W, Tang F (2016). Single-cell sequencing in stem cell biology. Genome Biol..

[2] Stegle O, Teichmann S, Marioni JC (2015). Computational and analytical challenges in single-cell transcriptomics. Nat. Rev. Genet..

[3] Stoeckius M (2017). Simultaneous epitope and transcriptome measurement in single cells. Nat. Methods.

[4] Papalexi E, Satija R (2018). Single-cell rna sequencing to explore immune cell heterogeneity. Nat. Rev. Immunol..

[5] Kolodziejczyk AA, Kim JK, Svensson V, Marioni JC, Teichmann SA (2015). The technology and biology of single-cell rna sequencing. Mol. Cell.

[6] Tusi BK (2018). Population snapshots predict early haematopoietic and erythroid hierarchies. Nature.

[7] Huang W, Cao X, Biase FH, Yu P, Zhong S (2014). Time-variant clustering model for understanding cell fate decisions. Proc. Nat. Acad. Sci..

[8] Barjoseph Z, Gitter A, Simon I (2012). Studying and modelling dynamic biological processes using time series gene expression data. Nat. Rev. Genet..

[9] Oh V, Li RW (2021). Temporal dynamic methods for bulk rna-seq time series data. Genes.

[10] Ding J, Sharon N, Bar-Joseph Z (2022). Temporal modelling using single-cell transciptomics. Nat. Rev. Genet..

[11] Whitfield M, Sherlock G, Saldanha AEA (2002). Identification of genes periodically expressed in the human cell cycle and their expressio in tumors. Mol. Biol. Cell.

[12] Muskovic W, Slavich E, Maslen B (2022). High temporal resolution rna-seq time course data reveals widespead synchronous activation between mammalian lncrnas and neighboring protein-coding genes. Genome Res..

[13] Magwene P, Lizardi P, Kim J (2003). Reconstructing the temporal ordering of biological samples using microarray data. Bioinformatics.

[14] Gupta A, Bar-Joseph Z (2008). Extracting dynamics from static cancer expression data. IEEE/ACM Trans. Comput. Biol. Bioinf..

[15] Zeisel, A., K$$\ddot{o}$$stler, W. & Moloski, e. a., N. Coupled pre-mrna and mrna dynamics unveil operational strategies underlying transcriptional responses to stimuli. *Mol. Syst. Biol.* **7**, 529 (2011). 10.1038/msb.2011.62 PMC3202801 21915116

[16] Saelens W, Cannoodt R, Todorov H, Saeys Y (2019). A comparison of single-cell trajectory inference methods. Nat. Biotechnol..

[17] Pratapa A, Jalihal AP, Law JN, Bharadwaj A, Murali TM (2020). Benchmarking algorithms for gene regulatory network inference from single-cell transcriptomic data. Nat. Methods.

[18] Langfelder P, Horvath S (2008). Wgcna: An r package for weighted correlation network analysis. BMC Bioinform..

[19] Chan T, Stumpf M, Babtie A (2017). Gene regulatory network inference from single-cell data using multivariate information measures. Cell Syst..

[20] Kim S (2015). ppcor: An r package for a fast calculation to semi-partial correlation coefficients. Commun. Stat. Appl. Methods.

[21] Huynh-Thu V, Irrthum A, Wehenke L, Geurts P (2010). Inferring regulatory networks from expression data using tree-based methods. PLoS ONE.

[22] Moerman T (2019). Grnboost2 and arboreto: Efficient and scalable inference of gene regulatory networks. Bioinformatics.

[23] Furchtgott LSM, Menon V, Ramanathan S (2017). Discovering sparse transcription factor codes for cell states and state transitions during development. Elife.

[24] Candes EJ, Tao T (2005). Decoding by linear programming. IEEE Trans. Inf. Theory.

[25] Tibshirani, R. Regression shrinkage and selection via the lasso. *J. Royal Statist. Soc. Series B (Methodological)* 267–288 (1996).

[26] Stock J, Watson M (2001). Vector autoregressions. J. Econ. Perspect..

[27] Friedman J, Hastie T, Tibshirani R (2008). Sparse inverse covariance estimation with the graphical lasso. Biostatistics.

[28] Blumensath T (2013). Compressed sensing with nonlinear observations and related nonlinear optimization problems. IEEE Trans. Inf. Theory.

[29] Chatfield, C. & Xing, H. *The Analysis of Time Series: An Introduction with R. 7th edition* (Chapman and Hall/CRC, 2019).

[30] Garrett Fitzmaurice, N. L. . J. W. *Applied Longitudinal Analysis, 2nd Edition* (Chapman and Hall/CRC, 2011).

[31] Chui, C. & Chen, G. *Kalman Filtering with Real-Time Applications* (Springer, 2017).

[32] Matsumoto H (2017). Scode: An efficient regulatory network inference algorithm from single-cell rna-seq during differentiation. Bioinformatics.

[33] Deshpande, A., Chu, L., Stewart, R. & Gitter, A. Network inference with granger causality ensembles on single-cell transcriptomic data. *Preprint at https://doi.org/10.1101/534834* (2020+). 10.1016/j.celrep.2022.110333 PMC9093087 35139376

[34] Aubin-Frankowski, P. & Vert, J. Gene regulation inference from single-cell rna-seq data with linear differential equations and velocity inference. *Preprint at https://doi.org/10.1101/464479* (2020+). 10.1093/bioinformatics/btaa576 33026066

[35] Carrizosa E, Olivares-Nadal A, Ramírez-Cobo P (2017). A sparsity-controlled vector autoregressive model. Biostatistics.

[36] Wilms, I., Basu, S., Bien, J. & Matteson, c. Sparse identification and estimation of large-scale vector autoregressive moving averages. *J. Am. Stat. Assoc.* **118:541**, 571–582 (2023). 10.1080/01621459.2021.1942013 PMC10281743 37346226

[37] Boyd S (2011). Distributed optimization and statistical learning via the alternating direction method of multipliers. Found. Trends Mach. Learn..

[38] Mooney, C. Z., Duval, R. D. & Duvall, R. *Bootstrapping: A nonparametric approach to statistical inference*. 94-95 (Sage, 1993).

[39] Bickel PJ, Levina E (2008). Covariance regularization by thresholding. Ann. Stat..

[40] Cai T, Liu W (2011). Adaptive thresholding for sparse covariance matrix estimation. J. Am. Stat. Assoc..

[41] Liu H, Wang L, Zhao T (2014). Sparse covariance matrix estimation with eigenvalue constraints. J. Comput. Graph. Stat..

[42] Fan J, Liao Y, Mincheva M (2013). Large covariance estimation by thresholding principal orthogonal complements. J. Royal Stat. Soc: Series B (Stat. Methodol.).

[43] Tian L (2020). Benchmarking single cell rna-sequencing analysis pipelines using mixture control experiments. Nat. Methods.

[44] Bingsheng H, Hai Y, Shengli W (2000). Alternating direction method with self-adaptive penalty parameters for monotone variational inequalities. J. Optim. Theory Appl..

[45] Shengli W, Lizhi L (2001). Decomposition method with a variable parameter for a class of monotone variational inequality problems. J. Optim. Theory Appl..

[46] Efron B (1979). Bootstrap methods: Another look at the jackknife. Ann. Stat..

[47] Guo G (2010). Resolution of cell fate decisions revealed by single-cell gene expression analysis from zygote to blastocyst. Dev. Cell.

[48] Chu LF (2016). Single-cell rna-seq reveals novel regulators of human embryonic stem cell differentiation to definitive endoderm. Genome Biol..

[49] Hayashi T (2018). Single-cell full-length total rna sequencing uncovers dynamics of recursive splicing and enhancer rnas. Nat. Commun..

[50] Xie X (2021). Single-cell transcriptomic landscape of human blood cells. Natl. Sci. Rev..

[51] Saiz N, Plusa B (2013). Early cell fate decisions in the mouse embryo. Reproduction.

[52] Nakai-Futatsugi Y, Niwa H (2015). Epiblast and primitive endoderm differentiation: Fragile specification ensures stable commitment. Stem Cell.

[53] Chazaud C, Yamanaka Y (2016). Lineage specification in the mouse preimplantation embryo. Development.

[54] Sozen B, Can A, Demir N (2014). Cell fate regulation during preimplantation development: A view of adhesion-linked molecular interactions. Dev. Biol..

[55] Rossant J, Tam P (2009). Blastocyst lineage formation, early embryonic asymmetries and axis patterning in the mouse. Development.

[56] Tenenbaum JB, de Silva V, Langford JC (2000). A global geometric framework for nonlinear dimensionality reduction. Science.

[57] Shannon P (2003). Cytoscape: A software environment for integrated models of biomolecular interaction networks. Genome Res..

[58] Frum T, Ralston A (2015). Cell signaling and transcription factors regulating cell fate during formation of the mouse blastocyst. Trends Genet..

[59] Menchero S, Rayon T, Andreu M, Manzanares M (2017). Signaling pathways in mammalian preimplantation development: Linking cellular phenotypes to lineage decisions. Dev. Dyn..

[60] Goolam M (2016). Heterogeneity in oct4 and sox2 targets biases cell fate in 4-cell mouse embryos. Cell.

[61] Zernicka-Goetz M, Morris S, Bruce A (2009). Making a firm decision: multifaceted regulation of cell fate in the early mouse embryo. Nat. Rev. Genet..

[62] Niakan *et al.* Sox17 promotes differentiation in mouse embryonic stem cells by directly regulating extraembryonic gene expression and indirectly antagonizing self-renewal. *Genes Dev.* 312–326 (2010). 10.1101/gad.1833510 PMC2811832 20123909

[63] Camacho DM, Collins KM, Powers RK, Costello JC, Collins JJ (2018). Next-generation machine learning for biological networks. Cell.

[64] Gao N, MinhazUd-Dean S, Gandrillon O, Gunawan R (2018). Sincerities: Inferring gene regulatory networks from time-stamped single cell transcriptional expression profiles. Bioinformatics.

[65] Sanchez-Castillo M, Blanco D, Tienda-Luna I, Carrion MC, Huang Y (2018). A bayesian framework for the inference of gene regulatory networks from time and pseudo-time series data. Bioinformatics.

[66] Hastie, T., Tibshirani, R. & Friedman, J. *The elements of statistical learning: data mining, inference, and prediction* (Springer Science & Business Media, 2009).

[67] Aibar S (2017). Scenic: Single-cell regulatory network inference and clustering. Nat. Methods.

[68] Qiu X (2020). Inferring causal gene regulatory networks from coupled single-cell expression dynamics using scribe. Cell Syst..

