# Supplementary material for: Unveiling gene regulatory networks during cellular state transitions without linkage across time points
Source: Sci Rep. 2024 May 29;14:12355. doi: 10.1038/s41598-024-62850-1 (PMC11137113; doi:10.1038/s41598-024-62850-1)
Supplement: Supplementary file 1 — Supplementary Information. [file 41598_2024_62850_MOESM1_ESM.pdf]

Supplementary Information for "Unveiling gene regulatory networks during cellular state transitions without linkage across time points"

May 28, 2024

1 Supplementary Methods

1.1 Solving COSLIR via ADMM

Eq. 3 in the main text represents a non-convex optimization problem, and we employed the ADMM method to solve it. The augmented Lagrangian form of COSLIR can be expressed as:

$$\begin{aligned}\mathcal{L}(A,B,C,\Pi_1,\Pi_2) =& \frac{||\hat{\Sigma}_{t+1} - B\hat{\Sigma}_t C^T||_F^2}{||\hat{\Sigma}_{t+1} - \hat{\Sigma}_t||_F^2} + \eta \frac{||\hat{\mu}_{t+1} - \frac{B+C}{2}\hat{\mu}_t||_2^2}{||\hat{\mu}_{t+1} - \hat{\mu}_t||_2^2} \\ &+\lambda||A||_1 + \langle B-C, \Pi_1\rangle + \frac{\rho}{2}||B-C||_F^2 \\ &+\langle A+I-\frac{B+C}{2}, \Pi_2\rangle + \frac{\rho}{2}||A+I-\frac{B+C}{2}||_F^2.\end{aligned} \quad (1)$$

1.2 Algorithm

The detailed description of the ADMM algorithm for solving COSLIR is given in Algorithm 1.

We also added some optimisation tricks into our published code to expedite the convergence rate of ADMM, a strategy proven effective in both simulation studies and real data experiments [1, 3].

1.3 A requirement for the successful application of COSLIR

We proposed a condition for the applicability of COSLIR based on empirical observations. As shown in Fig. S1a, a small Frobenius norm of the true A is necessary for optimal recovery. However, since the true value of A is typically unknown in real applications, we computed an alternative index:

$$r = \frac{||\Sigma_2 - \Sigma_1||_F}{||\Sigma_1||_F}. \quad (4)$$

Algorithm 1: ADMM for COSLIR

Input: $\hat{\Sigma}_t$, $\hat{\Sigma}_{t+1}$, $\hat{\mu}_t$, $\hat{\mu}_{t+1}$, η, λ, ρ
Output: The estimator $\hat{A}$
while $\max\{||\nabla_{A_n}\mathcal{L}||_F, ||\nabla_{B_n}\mathcal{L}||_F, ||B_n - C_n||_F, ||A_n + I - (B_n + C_n)/2||_F\} < \varepsilon$ **do**

Update B by solving $B_n = arg\min_B \mathcal{L}(A_{n-1}, B, C_{n-1}, \Pi_{n-1}^{(1)}, \Pi_{n-1}^{(2)})$;

Update C by solving $C_n = arg\min_C \mathcal{L}(A_{n-1}, B_n, C, \Pi_{n-1}^{(1)}, \Pi_{n-1}^{(2)})$;

Update A by solving $A_n = arg\min_A \mathcal{L}(A, B_n, C_n, \Pi_{n-1}^{(1)}, \Pi_{n-1}^{(2)})$;

Update $\Pi_n^{(1)}$, $\Pi_n^{(2)}$:

$$\Pi_n^{(1)} = \Pi_{n-1}^{(1)} + \rho(B_n - C_n) \tag{2}$$

$$\Pi_n^{(2)} = \Pi_{n-1}^{(2)} + \rho(A_n + I - \frac{B_n + C_n}{2}) \tag{3}$$

end
return A_n

While r may not perfectly represent $||A||_F$, they exhibited a high correlation, as depicted in Fig. S1b. We recommended that r should be less than 2; otherwise, additional normalization steps may be required before applying COSLIR.

2 Supplementary Results

2.1 Simulation Study

We conducted numerous simulation experiments to assess the capacity and performance of COSLIR, testing it in two scenarios: oracle cases and sample cases. In the oracle case, the true mean and covariance of each stage are known. In the sample case, the true mean and covariance of each stage are unknown, but we have independent samples from each stage, enabling us to estimate the mean and covariance.

Let A_0 be the oracle GRN, A the estimate of A_0, and $\Sigma_1, \Sigma_2, \mu_1, \mu_2$ be given to obtain A via COSLIR. We designed the following criteria to evaluate the

performance of COSLIR and guide the tuning of hyperparameters:

$$\begin{aligned} err &= \frac{||A_0 - A||_F}{||A_0||_F}, & (5)\\ e_\Sigma &= \frac{||\Sigma_2 - (I+A)\Sigma_1(I+A)^T||_F}{||\Sigma_2 - \Sigma_1||_F}, & (6)\\ e_\mu &= \frac{||\mu_2 - (I+A)\mu_1||_F}{||\mu_2 - \mu_1||_2}, & (7)\\ s_0 &= \frac{\#\{\text{non-zero elements of } A\}}{\#\{\text{elements of } A\}}, & (8)\\ loss &= e_\Sigma + \eta e_\mu. & (9) \end{aligned}$$

In both oracle and sample cases, we used e_Σ, e_μ, and s_0 for tuning λ and η, while *loss* was employed for tuning the clipping threshold in sample cases. Additionally, *err* measured the relative difference between the true A_0 and its estimator. The determined values of η and λ in Fig. 2 of the main text are summarized in Table S1 below.

We evaluated our proposed model selection criteria for determining the two tuning parameters, λ and η. The results proved to be robust concerning the value of η (see Fig. S2), and our criteria consistently helped identify optimal or suboptimal values of λ (see Fig. S3).

Tables S2 and S3 demonstrated how we tuned the crucial parameter λ in the oracle and sample cases, respectively, based on the criteria of e_Σ, e_μ, and s_0. The exact value of the estimator closely matched the correct ones generated for the simulation, as shown in Table S2, where *err* is on the order of 10^{-3}. Table S4 reveals that the clipping threshold should be set to 0.01 when considering s_0 and *loss* of $\hat{A}_t(\varepsilon)$ together.

In Fig. S3, we demonstrated the performance of COSLIR as the true sparsity of the interacting matrix varies in the oracle case. The more sparse the true interacting matrix, the better the performance. Fig. S4 illustrated how the performance of COSLIR varies with the clipping threshold and the confidence threshold. Precision increases with both thresholds, while recall decreases.

2.2 Real-Data analysis

2.2.1 More details on the RT-PCR data analysis

See Table S5 for the number of samples in each cell state. Figures S6 and S7 validated the results inferred by COSLIR. Here, we validated whether two genes have a reported regulatory interaction, regardless of whether it is activation or inhibition, as this information is rarely included in these databases.

The p values in Fig. S6 were calculated as the probability that, if the n non-vanishing elements inferred by COSLIR are uniformly sampled among the total N elements in the matrix A, at least m of them overlap with the total M

elements reported by at least one database. The formula for the p value is

$$\sum_{i=m}^{min(m,n)} \frac{C_M^i C_{N-M}^{n-i}}{C_N^n}.$$

It is known that embryonic development towards the EPI and PE stages share many common gene regulatory interactions and signaling pathways. Therefore, we used the ChIP-seq (ESC) database to validate the inferred GRN driving ICM towards PE, as the gene regulatory information directly measured for PE development or XEN cells is scarce.

Inference of A matrix from ICM towards EPI: The clipping threshold is 0.01, the confidence threshold is 0.8, and the rescaled value threshold is 0.25. Inference of A matrix from ICM towards PE: The clipping threshold is 0.01, the confidence threshold is 0.8, and the rescaled value threshold is 0.1.

2.2.2 More details on the scRNA-seq data analysis

We pre-processed the scRNA-Seq data following the workflow in BEELINE [2]. The pseudotime of the data was calculated using Slingshot [4], with cells measured at 0 h as the start cluster and cells measured at the end time point (72 h for mESC and 96 h for hESC) as the end cluster. We then selected genes that varied more over pseudotime. This was done using the general additive model implemented with the R package 'gam' to calculate both variance and P-value. The Bonferroni method was used to correct for multiple hypothesis testing. Namely, if the P-value is 0.01 with n hypotheses to be tested (n is the number of genes), we selected those genes that have a P-value less than $0.01/n$. Finally, all TFs (genes that appeared as nodes in the ground truth network) and the 500 most variable genes (with the highest variance) were added to our dataset.

We used the subnetwork in the database formed by all the selected genes as the ground truth network. In addition, for each network predicted by a GRN inference algorithm, we selected only those interactions originating from a TF for further evaluation. Furthermore, since only adjacent time steps were considered in our paper, some cells might have near-zero expressions in certain pairs of time steps, which would lead to errors in SINCERITES. So we sometimes deleted these cells to ensure the feasibility of the algorithms.

After comparing the loss and sparsity with $\eta \in 10^{-4}, 10^{-2}, 1, 10^2, 10^4$, we saw that the performance was robust to the choice of η. So we fixed $\eta = 5$ throughout the real data analysis. For the parameters λ and clipping threshold, their performance on each dataset was compared in Figures S8, S9, S10, S11, where we chose $\lambda \in 10^{-3}, 10^{-4}, 10^{-5}, 10^{-6}, 10^{-7}$ and clipping threshold in $5 \times 10^{-4}, 0.001, 0.005, 0.01, 0.05, 0.1$. We can see that the errors e_Σ, e_μ decrease with respect to $-\log\lambda$ and reach a stable value around $\lambda = 10^{-5}$. The inferred matrix was sparser with larger λ, and we noticed in practice that an output that is too sparse (e.g. when $\lambda = 10^{-5}$) typically did not have good performance

on EPR (since EPR's selection of the most important edges has a similar effect to the sparseness penalty). Therefore, we used $\lambda = 10^{-6}$ throughout the analysis of the scRNA-Seq datasets. In these figures, we can see that both loss and sparsity increase with the clipping threshold and have little variation below 0.01. Therefore, we used a clipping threshold of 0.01 to obtain the most sparse matrix with an acceptable loss throughout the analysis. In all experiments, we bootstrap 50 times.

Figures S12 and S13 showed the proportions of database-supported gene regulatory relationships predicted by each method out of all database-validated predictions in mouse and human scRNA-seq datasets. Very little overlap was found, suggesting that COSLIR should be a useful complement to existing methods.

3 Supplementary Figures

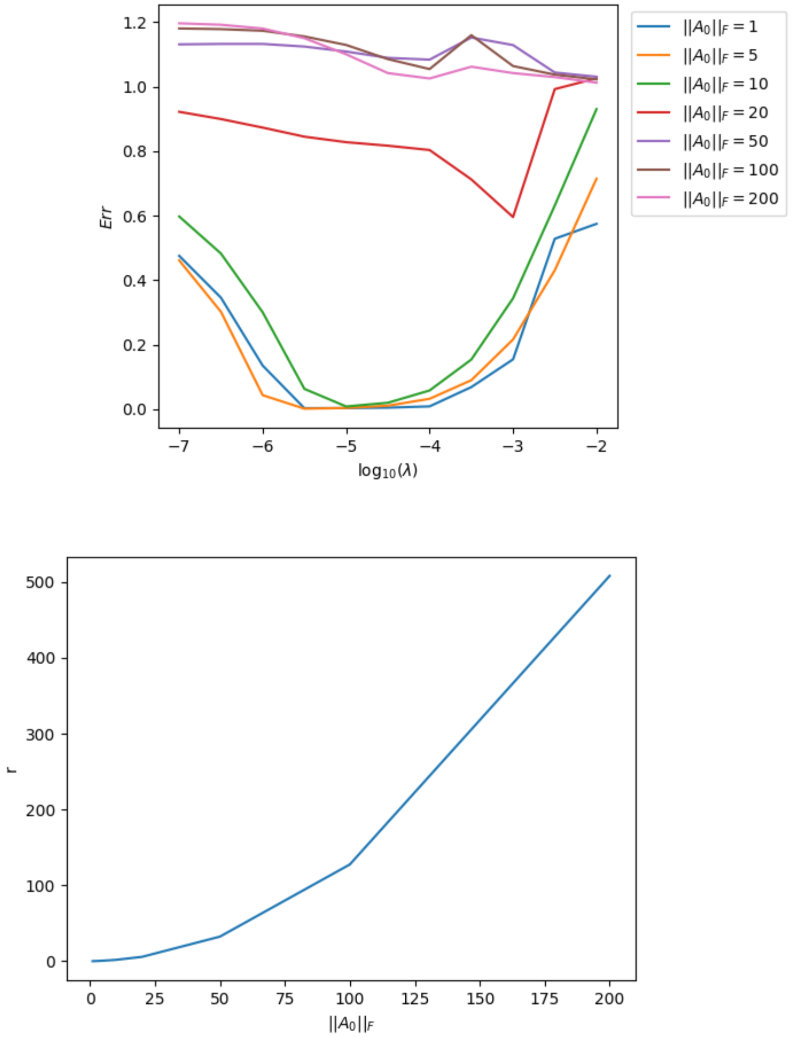

Figure S1: (a) $err = \frac{||A_0 - A_1||_F}{||A_0||_F}$ versus the tuning parameter λ within different scales of the true A (denoted as A_0). The dimension of the data is fixed at 100. Each line shows the average of 50 independent results. (b) Correlation between the norm of A_0 and the index r. All results are from the oracle cases in the simulation study.

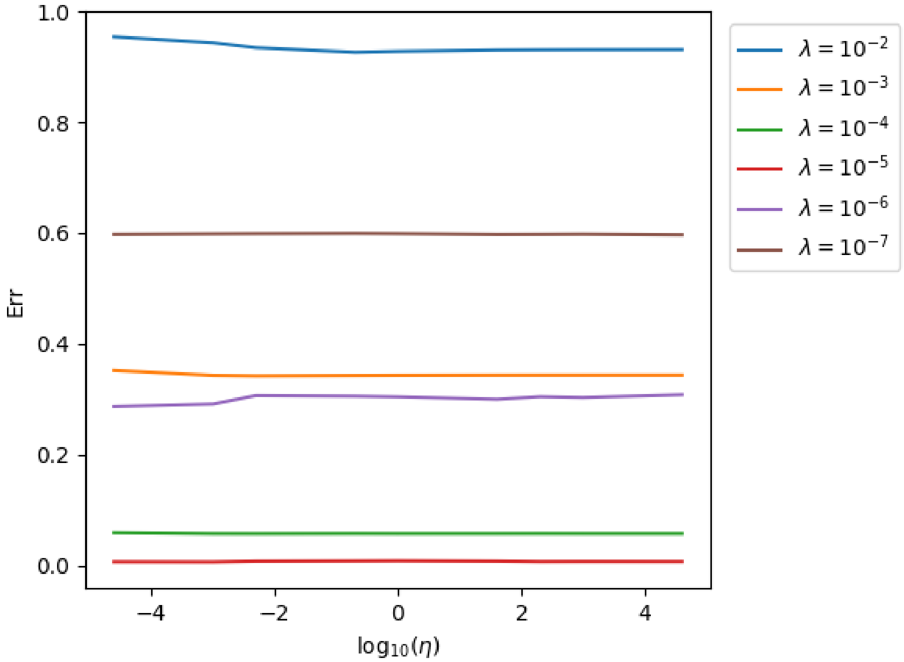

Figure S2: η versus err with different λ in the oracle cases.

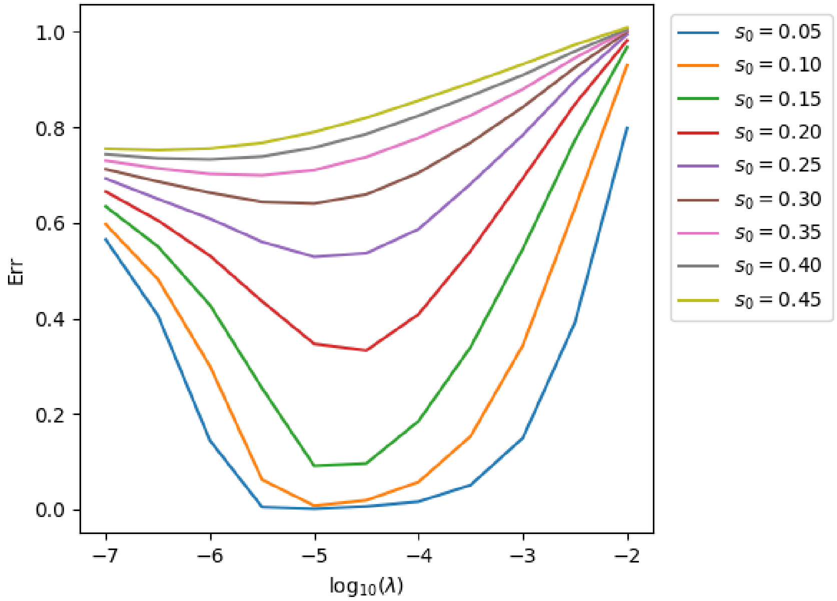

Figure S3: Sparsity of A_0 versus λ in the oracle cases. The dimension is set to 100 and the norm of A_0 is set to 10.

(a) Sample size is 1000 **(b)** Sample size is 5000

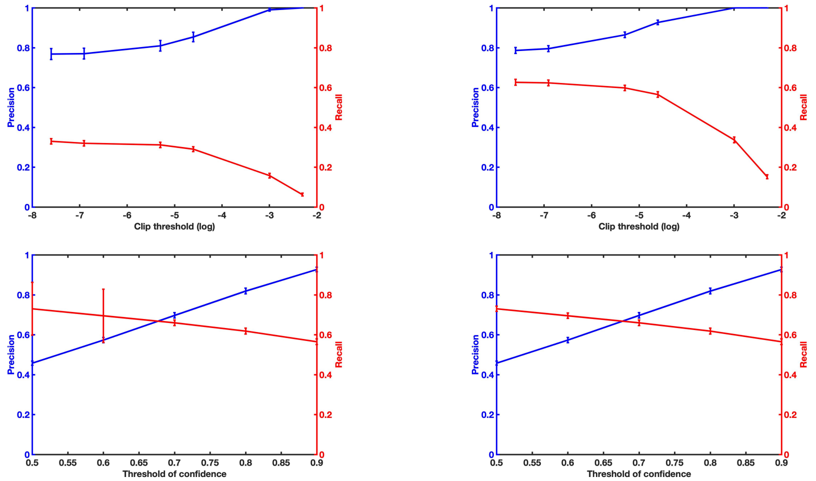

Figure S4: Precision and recall vary with clipping threshold and confidence threshold. In the left two figures, the confidence is held constant at 0.9, while in the right two figures, the clipping threshold is fixed at 0.01. The dimension is 100. These figures represent the average outcomes from 50 independent trials.

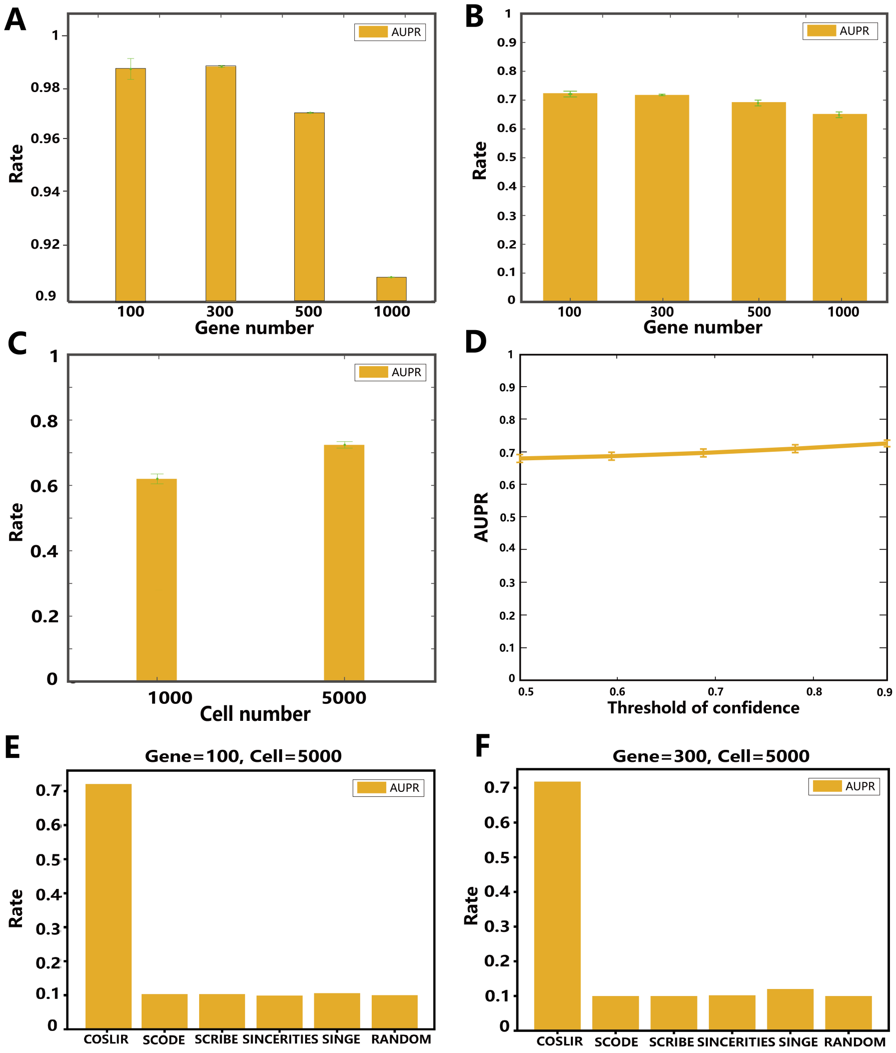

Figure S5: The values of AUC in Fig. 2 of the main text.

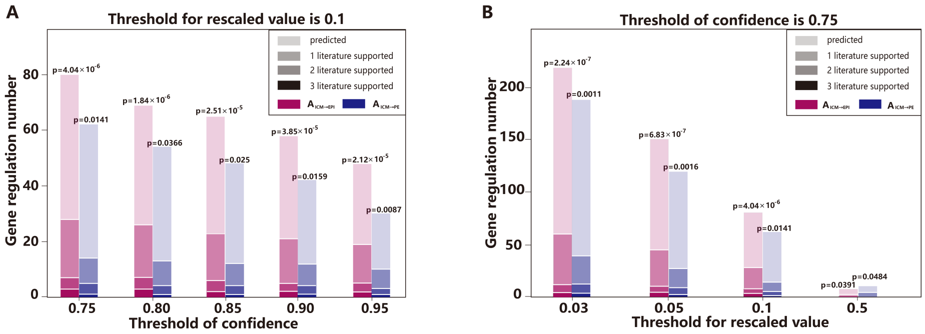

Figure S6: The regulatory network identification results from COSLIR on the RT-PCR dataset are summarized, considering different confidence thresholds and rescaled values. The number of regulations with literature support and the p-value for our predictions are provided. Panel (A) showcases the impact of various confidence thresholds while holding the rescaled value threshold constant at 0.1. In (B), we explore different thresholds on the rescaled values, maintaining a fixed confidence threshold of 0.75.

$A_{ICM\rightarrow EPI}$ verified by databases

Regulation genes	Rescaled value	Confidence	ChIP-atlas	BioGRID	TRRUST
GATA3 → KLF4	0.25149	0.98605	✓		
GATA4 ⊣ GATA6	-1.1489	0.99767		✓	
GATA4 → KLF4	0.38715	0.99535	✓		
GATA6 → SOX13	1.02739	0.95581	✓		
GATA6 → ESRRB	0.32978	0.97674	✓		
KLF4 → KLF4	1.39201	1	✓		
KRT8 → KLF4	0.46228	1	✓		
NANOG → KLF4	0.77241	0.93953	✓		✓
NANOG → SOX2	0.36288	0.99767	✓	✓	
PDGFA → KLF4	0.6532	1	✓		
PDGFA → SOX13	0.43976	0.96279	✓		
PDGFA → POU5F1	0.27996	1	✓		
PDGFA → SOX2	0.26044	1	✓		
PDGFRA → SOX13	0.71753	0.97442	✓		
PDGFRA → POU5F1	0.39495	1	✓		
POU5F1 ⊣ SNAIL	-0.7538	0.99535	✓	✓	
POU5F1 → SOX13	0.52616	0.85349	✓	✓	✓
POU5F1 → SOX2	0.516	1	✓	✓	✓
POU5F1 → POU5F1	0.42345	0.99535	✓	✓	✓
POU5F1 ⊣ KLF5	-0.3537	0.96744	✓	✓	✓
SALL4 → SOX13	1.43285	0.92558	✓		
SALL4 → KLF4	1.15618	1	✓		
SALL4 → POU5F1	0.29483	1	✓		
SOX17 → TSPAN8	0.67657	0.86279	✓		
SOX2 ⊣ GRHL2	-2.677	0.99535		✓	
SOX2 ⊣ KLF5	-1.2234	0.99767	✓		
SOX2 → SOX2	0.85517	1	✓		✓
SOX2 ⊣ KLF4	-0.7845	0.90698	✓		
SOX2 ⊣ NANOG	-0.301	0.98372	✓	✓	
TCFAP2C → POU5F1	0.4643	1	✓	✓	
TCFAP2C → SOX13	0.43457	0.85814	✓		

$A_{ICM\rightarrow PE}$ verified by databases

Regulation genes	Rescaled value	Confidence	ChIP-atlas	BioGRID	TRRUST
GATA4 ⊣ GATA6	-3.92724	0.990964		✓	
SOX2 ⊣ KLF4	-0.34557	0.966867	✓		
SOX2 ⊣ SOX2	-0.30131	1	✓		✓
NANOG ⊣ SOX13	-0.25893	0.936747	✓	✓	✓
SOX2 ⊣ ESRRB	-0.2504	1	✓	✓	
KLF4 ⊣ KLF4	-0.23347	1	✓		
POU5F1 → POU5F1	0.215396	0.951807	✓	✓	✓
SALL4 → POU5F1	0.207051	0.933735	✓		
NANOG ⊣ NANOG	-0.20454	1	✓	✓	
SOX2 ⊣ NANOG	-0.20088	1	✓	✓	
POU5F1 ⊣ TCFAP2C	-0.2002	0.942771		✓	
SALL4 → SALL4	0.185147	0.996988			✓
POU5F1 ⊣ KLF4	-0.18244	0.903614	✓	✓	
NANOG ⊣ KLF4	-0.1802	0.873494	✓		✓
GATA4 ⊣ POU5F1	-0.17944	0.918675	✓		
SOX2 ⊣ KLF5	-0.17911	0.894578	✓		
GATA6 ⊣ ESRRB	-0.15283	0.990964	✓		
SOX2 → GATA4	0.147944	1	✓		
GATA6 → GATA4	0.122336	1		✓	
TCFAP2C → POU5F1	0.118665	0.933735	✓	✓	
NANOG ⊣ SOX2	-0.11699	0.996988	✓	✓	
POU5F1 ⊣ NANOG	-0.11288	0.996988	✓	✓	
PDGFA → POU5F1	0.104264	0.990964	✓		
POU5F1 ⊣ SOX2	-0.10251	0.996988	✓	✓	✓
GATA4 ⊣ MSC	-0.10056	0.894578			✓

$A_{ICM\rightarrow EPI}$ verified exclusively by recent literature

Regulation genes	Rescaled value	Confidence	ChIP-atlas	BioGRID	TRRUST	Recent litertature
GATA3 ⊣ GATA6	-0.6278	0.9093	✗	✗	✗	✓

$A_{ICM\rightarrow PE}$ verified exclusively by recent literature

Regulation genes	Rescaled value	Confidence	ChIP-atlas	BioGRID	TRRUST	Recent litertature
SOX17 ⊣ GATA6	-0.29245	0.8464	✗	✗	✗	✓

Figure S7: These tables provide a comprehensive overview of all identified edges in our prediction network obtained through COSLIR on the RT-PCR dataset. These edges are further corroborated by evidence from the ChIP-atlas, BioGRID, and TRRUST databases. Additionally, we cross-reference these findings with recently published literature to validate edges associated with cell state transitions[6, 5, 8, 7].

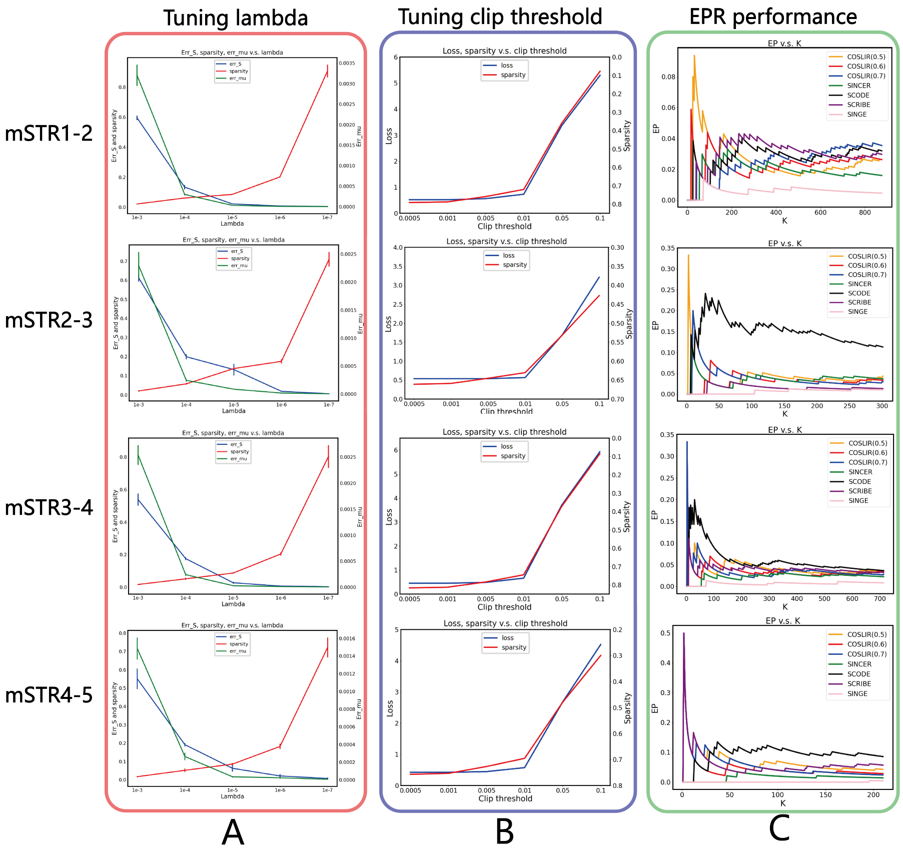

Figure S8: The performance evaluation of COSLIR using different values of λ and clipping thresholds on diverse single-cell RNAseq datasets, each comprising data from two consecutive time steps from mSTR (mESC + STRING network), and the Early Precision Rate (EPR) performance for various algorithms. A:The metrics e_Σ (err_S), e_μ (err_mu), and s_0 (sparsity) vary with respect to different values of λ (lambda). e_Σ and s_0 are presented on the same scale (left), while e_μ is displayed on a separate scale (right). The figures represent averages over 50 independent trials. B: Loss ($e_\Sigma + \eta e_\mu$) and s_0 vary with respect to the clipping threshold (no confidence truncation). C: Comparative performance of EPR for different algorithms (SINCERITIES, SCODE, SINGE, and COSLIR with different confidence clipping) across various datasets. This information can also aid in tuning the confidence threshold.

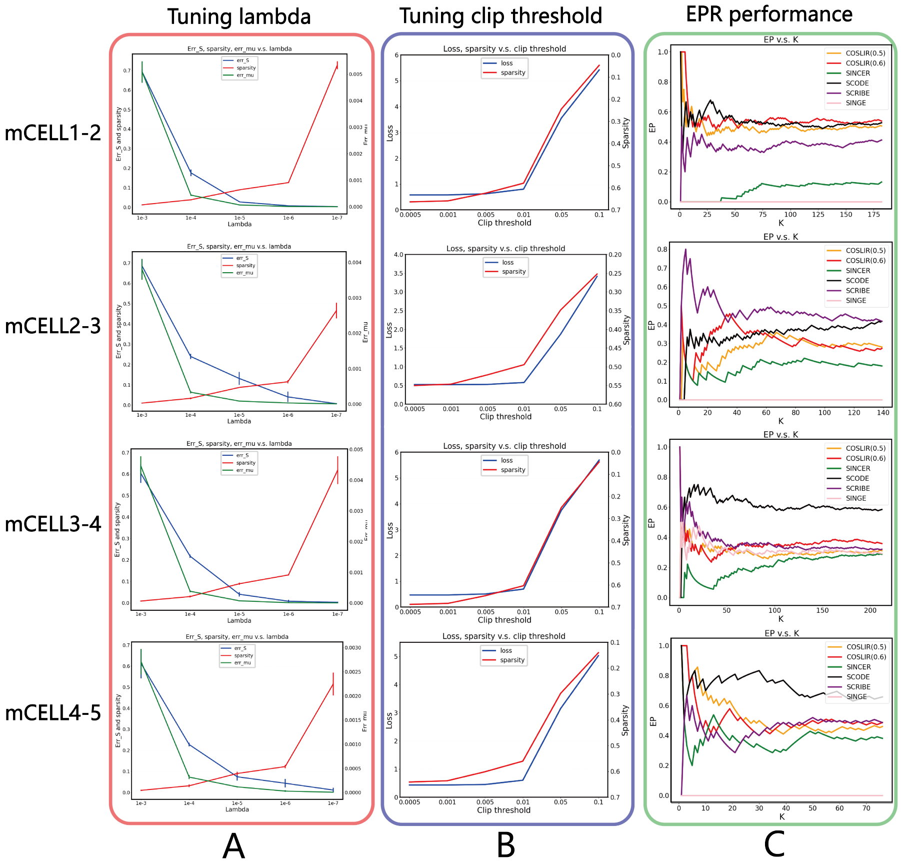

Figure S9: The performance evaluation of COSLIR using different values of λ and clipping thresholds on diverse single-cell RNAseq datasets, each comprising data from two consecutive time steps from mCELL (mESC + cell type-specific ChIP-Seq network), and the Early Precision Rate (EPR) performance for various algorithms. A:The metrics e_Σ (err_S), e_μ (err_mu), and s_0 (sparsity) vary with respect to different values of λ (lambda). e_Σ and s_0 are presented on the same scale (left), while e_μ is displayed on a separate scale (right). The figures represent averages over 50 independent trials. B: Loss ($e_\Sigma + \eta e_\mu$) and s_0 vary with respect to the clipping threshold (no confidence truncation). C: Comparative performance of EPR for different algorithms (SINCERITIES, SCODE, SINGE, and COSLIR with different confidence clipping) across various datasets. This information can also aid in tuning the confidence threshold.

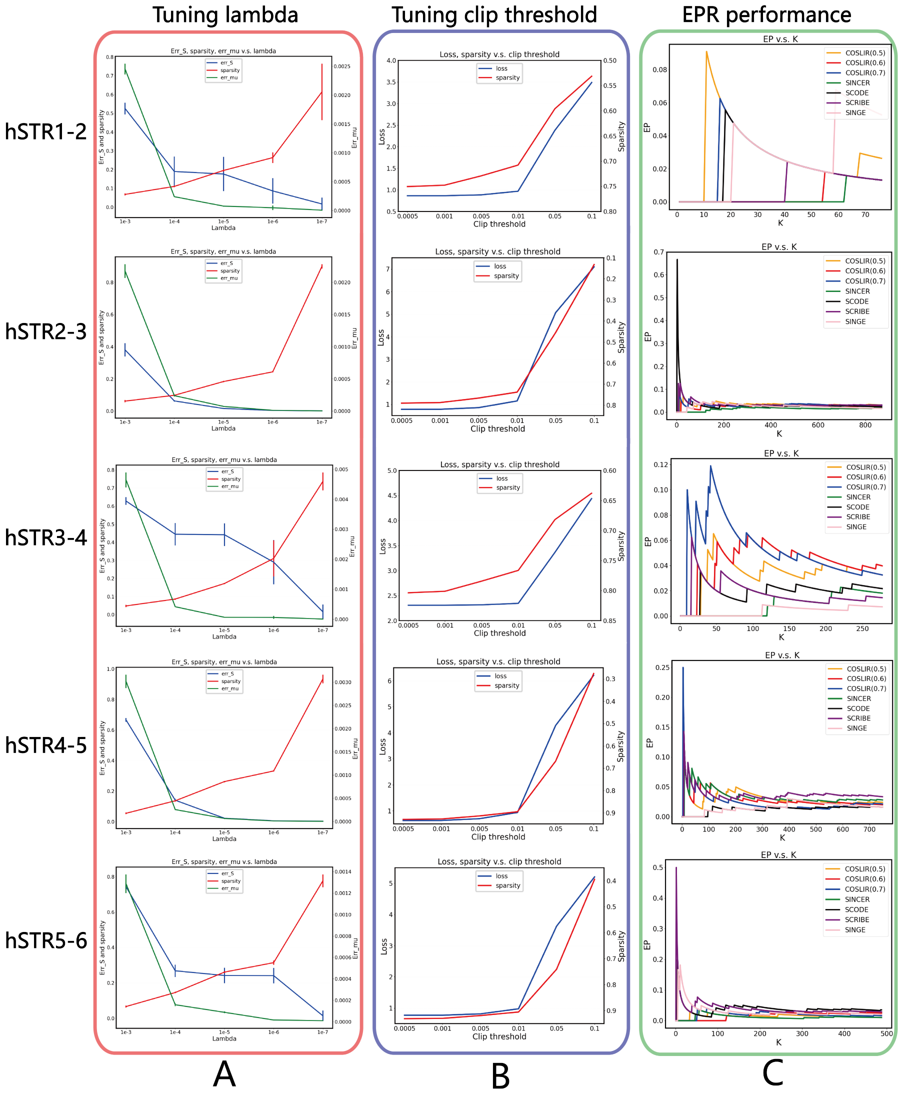

Figure S10: The performance evaluation of COSLIR using different values of λ and clipping thresholds on diverse single-cell RNAseq datasets, each comprising data from two consecutive time steps from hSTR (hESC + STRING network), and the Early Precision Rate (EPR) performance for various algorithms. A:The metrics e_Σ (err_S), e_μ (err_mu), and s_0 (sparsity) vary with respect to different values of λ (lambda). e_Σ and s_0 are presented on the same scale (left), while e_μ is displayed on a separate scale (right). The figures represent averages over 50 independent trials. B: Loss ($e_\Sigma + \eta e_\mu$) and s_0 vary with respect to the clipping threshold (no confidence truncation). C: Comparative performance of EPR for different algorithms (SINCERITIES, SCODE, SINGE, and COSLIR with different confidence clipping) across various datasets. This information can also aid in tuning the confidence threshold.

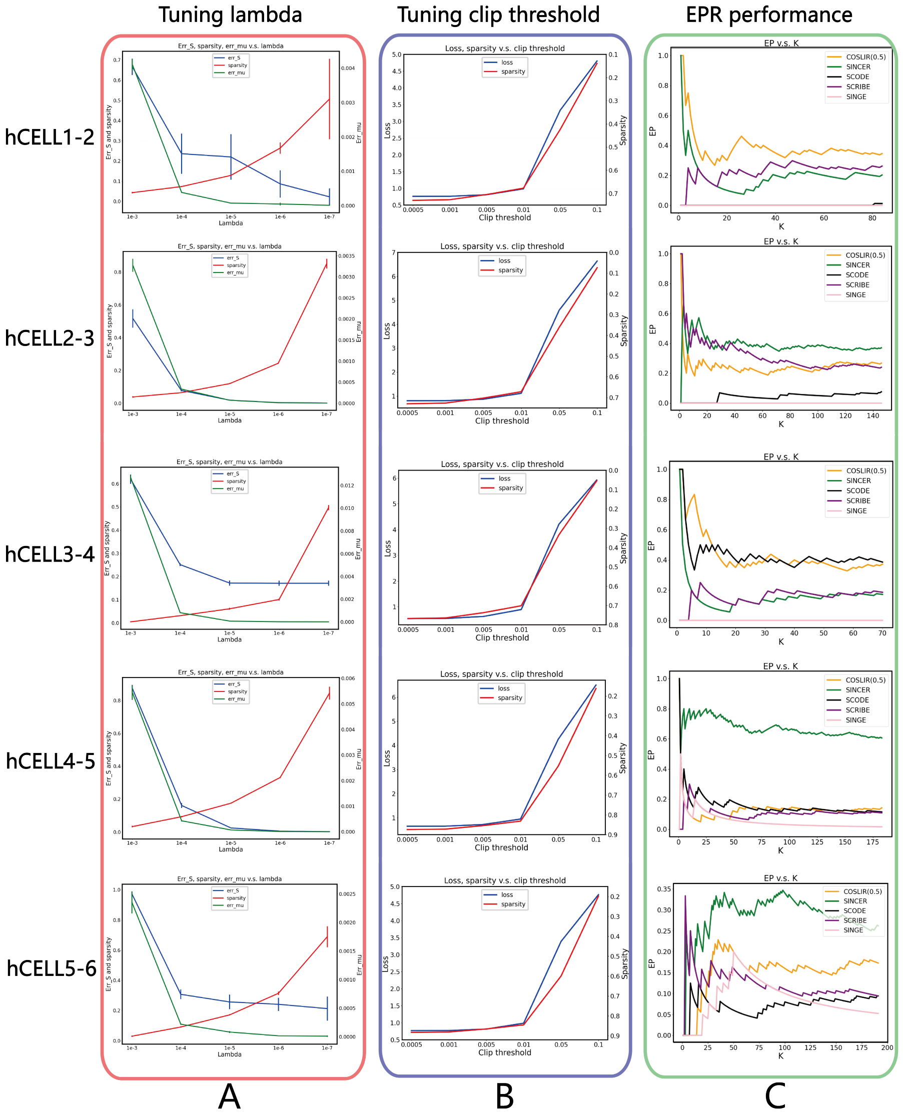

Figure S11: The performance evaluation of COSLIR using different values of λ and clipping thresholds on diverse single-cell RNAseq datasets, each comprising data from two consecutive time steps from hCELL (hESC + cell type-specific ChIP-Seq network), and the Early Precision Rate (EPR) performance for various algorithms. A:The metrics e_Σ (err_S), e_μ (err_mu), and s_0 (sparsity) vary with respect to different values of λ (lambda). e_Σ and s_0 are presented on the same scale (left), while e_μ is displayed on a separate scale (right). The figures represent averages over 50 independent trials. B: Loss ($e_\Sigma + \eta e_\mu$) and s_0 vary with respect to the clipping threshold (no confidence truncation). C: Comparative performance of EPR for different algorithms (SINCERITIES, SCODE, SINGE, and COSLIR with different confidence clipping) across various datasets. This information can also aid in tuning the confidence threshold.

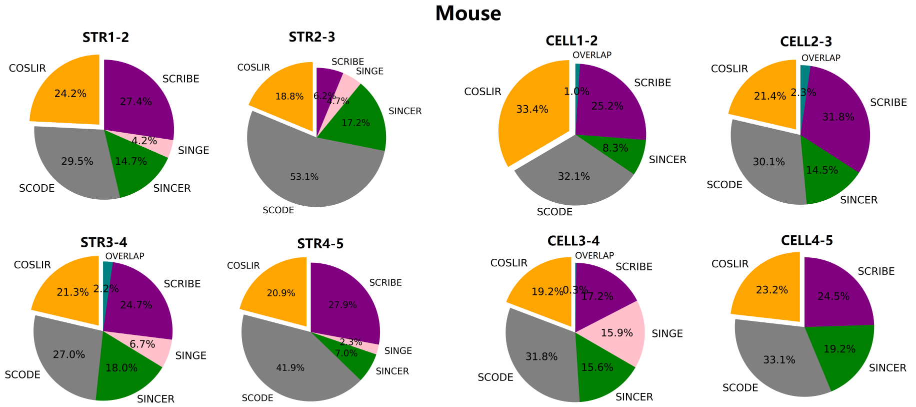

Figure S12: Pie charts depicting the distribution of predictions from various algorithms applied to mouse ESC datasets with 5 time steps, utilizing either STRING or cell-type-specific network as the ground truth[2].

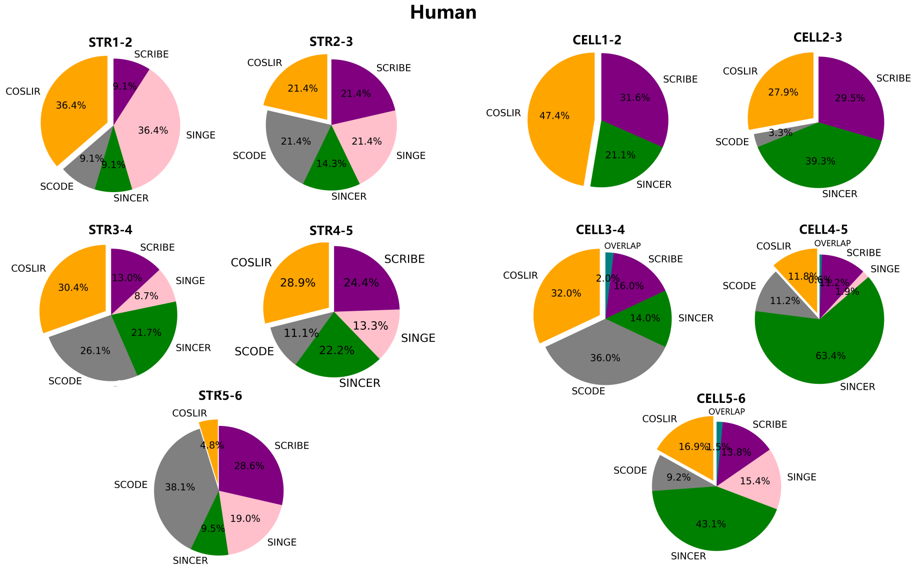

Figure S13: Pie charts depicting the distribution of predictions from various algorithms applied to human ESC datasets with 6 time steps, utilizing either STRING or cell-type-specific network as the ground truth[2].

Top 10 Edges Predicted by COSLIR

HSC→B

Source	Target
SOX4 (B)	RGS1 (B)
SOX4 (B)	AHSP (E)
SMC3 (H/B/E)	ADD3 (H/B)
EGR1 (H/B)	RGS1 (B)
TCF4 (H/B/E)	FOXP1 (B)
TCF4 (H/B/E)	MEF2C (H/B)
TCF4 (H/B/E)	AHSP (E)
FOXP1 (H/B)	FOXP1 (B)
RBPJ (H/B)	MEF2C (H/B)
TCF4 (H/B/E)	CRELD2 (B)

HSC→Erythrocyte

Source	Target
BRCA1 (E)	TYMS (H/B/E/M)
E2F2 (B/E)	TYMS (H/B/E/M)
SOX4 (B)	MPC2 (H/E)
GATA1 (E)	MS4A3 (H/E)
BRCA1 (E)	PIM1 (E)
SOX4 (B)	S100A4 (H/E/M)
GATA1 (E)	PIM1 (E)
SOX4 (B)	TMEM14B (E)
TCF4 (H/B/E)	MPC2 (H/E)
KLF1 (E)	MS4A3 (H/E)

HSC→Monocyte

Source	Target
KLF1 (E)	AIF1 (H/M)
SOX4 (B)	CDCA7 (H/M)
MYC (H)	GLTSCR2 (H/M)
EBF1 (B)	AIF1 (H/M)
HOXA9 (H)	CSTB (M)
MYC (H)	SEC63 (H)
TCF4 (H/B/E)	ITM2C (H/B/M)
MYC (H)	ITM2C (H/B/M)
HOXA9 (H)	CAPG (H/M)
GATA2 (H)	EREG (H/M)

Figure S14: COSLIR inferred top 10 gene-regulatory interactions in human blood single-cell datasets.

4 Supplementary Tables

Table S1: Hyper-parameters used in the numerical experiments presented in Fig. 2 of the main text

Sub-figure	case	Sample Size	Dimension	λ	η	clipping threshold	Confidence
a	oracle	-	100	10^{-5}	5	-	-
a	oracle	-	200	10^{-6}	5	-	-
a	oracle	-	300	10^{-6}	5	-	-
a	oracle	-	400	10^{-6}	5	-	-
a	oracle	-	500	10^{-6}	5	-	-
b	sample	1000	100	10^{-5}	5	0.01	0.9
b	sample	5000	100	10^{-5}	5	0.01	0.9
c	sample	5000	100	10^{-5}	5	0.01	0.9
c	sample	5000	300	10^{-5}	5	0.01	0.9
d	sample	5000	100	10^{-5}	5	0.01	0.9

Table S2: Tuning λ in the oracle cases. The criterion values are computed in a single experiment, with bold values corresponding to the values used in Fig. 2 of the main text. The simulation procedure is described in the Materials and Methods of the main text.

Dimension	λ	e_Σ	e_μ	s_0	err
100	10^{-2}	0.399	7.48×10^{-3}	0.093	0.588
	10^{-3}	0.059	5.671×10^{-4}	0.183	0.146
	10^{-4}	6.628×10^{-3}	5.379×10^{-5}	0.210	0.019
	$\mathbf{10^{-5}}$	$\mathbf{7.318 \times 10^{-4}}$	$\mathbf{8.486 \times 10^{-6}}$	**0.245**	$\mathbf{2.439 \times 10^{-3}}$
	10^{-6}	1.167×10^{-4}	1.509×10^{-6}	0.359	9.497×10^{-4}
	10^{-7}	1.164×10^{-4}	3.459×10^{-7}	0.694	0.435
200	10^{-2}	0.851	0.026	0.024	0.923
	10^{-3}	0.195	1.325×10^{-3}	0.140	0.369
	10^{-4}	0.025	1.171×10^{-4}	0.206	0.069
	10^{-5}	2.611×10^{-3}	1.214×10^{-5}	0.217	7.641×10^{-3}
	$\mathbf{10^{-6}}$	$\mathbf{2.962 \times 10^{-4}}$	$\mathbf{2.523 \times 10^{-6}}$	**0.258**	$\mathbf{1.100 \times 10^{-3}}$
	10^{-7}	3.359×10^{4}	5.006×10^{-7}	0.678	0.441
300	10^{-2}	0.975	0.049	8.677×10^{-3}	1.001
	10^{-3}	0.375	2.235×10^{-3}	0.093	0.569
	10^{-4}	0.054	1.738×10^{-4}	0.191	0.134
	10^{-5}	5.866×10^{-3}	1.665×10^{-5}	0.212	0.016
	$\mathbf{10^{-6}}$	$\mathbf{6.039 \times 10^{-4}}$	$\mathbf{2.397 \times 10^{-6}}$	**0.222**	$\mathbf{1.793 \times 10^{-3}}$
	10^{-7}	6.320×10^{-4}	4.277×10^{-7}	0.666	0.445
400	10^{-2}	0.985	0.083	6.768×10^{-3}	1.008
	10^{-3}	0.563	3.725×10^{-3}	0.057	0.724
	10^{-4}	0.090	2.352×10^{-4}	0.175	0.020
	10^{-5}	0.010	2.198×10^{-5}	0.211	0.029
	$\mathbf{10^{-6}}$	$\mathbf{1.057 \times 10^{-3}}$	$\mathbf{2.374 \times 10^{-6}}$	**0.218**	$\mathbf{3.145 \times 10^{-3}}$
	10^{-7}	9.939×10^{-4}	3.739×10^{-7}	0.653	0.445
500	10^{-2}	1.003	0.116	4.056×10^{-3}	1.012
	10^{-3}	0.751	6.606×10^{-3}	0.029	0.854
	10^{-4}	0.132	3.153×10^{-4}	0.157	0.280
	10^{-5}	0.015	2.852×10^{-5}	0.204	0.042
	$\mathbf{10^{-6}}$	$\mathbf{1.616 \times 10^{-3}}$	$\mathbf{3.029 \times 10^{-6}}$	**0.212**	$\mathbf{4.603 \times 10^{-3}}$
	10^{-7}	1.429×10^{-3}	5.390×10^{-7}	0.650	0.459

Table S3: Tuning λ in the sample cases (Dimension=100), using the average performance of 50 independent trials, with bold values corresponding to the values used in Fig. 2 of the main text. The simulation procedure is described in the Materials and Methods of the main text. $\eta = 5$. $\lambda = 10^{-5}$ is a reasonable choice based on the criterion described in the Materials and Methods of the main text, solely based on e_Σ, e_μ and s_0 before the clipping thresholding procedure, while the performance (Precision and Recall) for each λ is obtained after choosing the optimal clipping threshold, which happens to be always 0.01, as obtained in Tab. S4.

Dimension	Sample size	λ	e_Σ	e_μ	s_0	Precision	Recall
100	1000	10^{-3}	0.059	5.617×10^{-4}	0.183	0.840	0.221
		10^{-4}	6.628×10^{-3}	5.379×10^{-5}	0.210	0.832	0.313
		$\mathbf{10^{-5}}$	$\mathbf{7.318 \times 10^{-4}}$	$\mathbf{8.486 \times 10^{-6}}$	**0.245**	**0.818**	**0.306**
100	5000	10^{-3}	0.057	6.614×10^{-4}	0.191	0.929	0.499
		10^{-4}	6.417×10^{-3}	6.539×10^{-5}	0.220	0.931	0.568
		$\mathbf{10^{-5}}$	$\mathbf{7.031 \times 10^{-4}}$	$\mathbf{9.464 \times 10^{-6}}$	**0.259**	**0.926**	**0.592**
300	5000	10^{-3}	0.375	2.235×10^{-3}	0.093	0.856	0.225
		10^{-4}	0.054	1.738×10^{-4}	0.191	0.903	0.453
		$\mathbf{10^{-5}}$	$\mathbf{5.866 \times 10^{-3}}$	$\mathbf{1.665 \times 10^{-5}}$	**0.212**	**0.908**	**0.543**

Table S4: Tuning clipping threshold in the sample cases (Dimension=100). Criterion values are computed using the average performance of 50 independent trials, with bold values corresponding to those used in Fig. 2 of the main text. The simulation procedure is described in the Materials and Methods of the main text. $\lambda = 10^{-5}$ and $\eta = 5$.

Dimension	Sample size	clipping threshold	*loss*	Sparsity	Precision	Recall
100	1000	5×10^{-4}	0.369	0.099	0.706	0.351
		10^{-3}	0.369	0.098	0.713	0.348
		5×10^{-3}	0.369	0.095	0.770	0.326
		**0.01**	**0.370**	**0.091**	**0.818**	**0.306**
		0.05	0.389	0.062	0.981	0.161
		0.1	0.48	0.031	1.000	0.060
100	5000	5×10^{-4}	0.103	0.099	0.752	0.658
		10^{-3}	0.103	0.099	0.764	0.654
		5×10^{-3}	0.103	0.095	0.864	0.626
		**0.01**	**0.103**	**0.091**	**0.926**	**0.592**
		0.05	0.131	0.059	1.000	0.332
		0.1	0.288	0.030	1.000	0.153
300	5000	5×10^{-4}	0.123	0.130	0.781	0.606
		10^{-3}	0.123	0.106	0.790	0.603
		5×10^{-3}	0.123	0.095	0.850	0.575
		**0.01**	**0.123**	**0.091**	**0.908**	**0.543**
		0.05	0.151	0.061	0.999	0.321
		0.1	0.313	0.031	1.000	0.137

Table S5: Number of single-cell RT-PCR samples in each cell state

**Clusters**	8-cell	16-cell inner	16-cell outer	ICM	TE(morula)	EPI	PE	TE(blastocyst
**Sample number**	44	31	28	48	57	17	38	95

Table S6: TFs involved in the top-10 edges predicted by COSLIR in the three developmental processes in single cell human blood atlas. Use H for HSC, B for B cells, E for erythrocyte and M for monocyte in the 2nd column.

TF	Cell types involved	Annotation
BRCA1	H/B	Balance between quiescence and proliferation of HSCs [9].<br>Maintain genomic stability and affect HSC function [28].
E2F2	B/E	E2F2 mutant mice show erythroid maturation defects;<br>E2F1/E2F2 knockout B-cell maturation is defective [16].<br>Mediators for retinoblastoma, which is important in erythropoiesis [13].
EBF1	B	Important in B-lineage progenitors [31, 25].
EGR1	H/B	Block B cell apoptosis in immature B cells [21].<br>Maintain HSC number and function via regulation [19].
FOXP1	H/B	Repress SIPR2 in activated and germinal center B-cell [11].<br>Support HSC cell growth with a novel pathway [22].
KLF1	E	Hematopoietic-specific TF for erythroid, cooperated with GATA1 [10].<br>Enhance erythroid commitment and differentiation [30].
GATA1	E	Important in erythroid development and maturation [25, 23].
GATA2	H	Required for both primitive and definitive hematopoiesis [25].<br>GATA2 knockout leads to cell-autonomous loss of adult HSC [18].
HOXA9	H/B	Activated in HSC and repressed upon differentiation [24].<br>Help produce lymphoid precursors in the bone marrow [25].
MYC	H	N-Myc/c-Myc are important for HSC differentiation [25].
RBPJ	H/B	Downstream gene for NOTCH pathway. NOTCH:<br>affect HSC proliferation and their response to stress [15].<br>required for T/B lymphoid differentiation and cell-fate decision [20].
SOX4	B	Required for the survival of pro-B cell [17, 27].
SMC3	H/B	Facilitate B cell to plasma cell switch [26].<br>Necessary for for HSC functions and normal hematopoiesis [29].
TCF4	H/B/E	Down-regulated in HSCs,<br>involved in the development of B/T cells as activators,<br>promotes erythroid development [12, 14].

Table S7: Marker genes involved in the top-10 edges (targeted by TFs) predicted by COSLIR in the three developmental processes in single cell human blood atlas. Use H for HSC, B for B cells, E for erythrocyte and M for monocyte.

Marker genes	Cell types	Sub cell-types
ADD3	H/B	H: BNK, MLP<br>B: Immature B, Naive B, Regulatory B
AHSP	E	Erythrocyte
AIF1	H/M	H: GMP, HSC, LMPP, MLP, MPP<br>M: cMOP, Classical monocyte, hMDP,<br>Intermediate monocyte, Non-classical monocyte, Pre-monocyte
CAPG	H/M	H: GMP, LMPP, MLP<br>M: Classical monocyte, hMDP, Pre-monocyte
CDCA7	H/M	H: CMP, GMP, LMPP<br>M: cMOP, hMDP
CRELD2	B	Memory B, Plasma
CSTB	M	Intermediate monocyte, Non-classical monocyte
EREG	H/M	H: CMP, GMP, LMPP, MEP, MPP<br>M: cMOP, Classical monocyte, hMDP, Pre-monocyte
FOXP1	B	Immature B, Naive B, Pre-B, Regulatory B
GLTSCR2	H/M	H: CMP, MEP<br>M: cMOP, hMDP
ITM2C	H/B/M	H: GMP, LMPP<br>B: Memory B, Plasma<br>M: cMOP, hMDP
MEF2C	H/B	H: BNK, MLP<br>B: Immature B, Memory B, Naive B,<br>Plasma, Pre-B, Pro-B, Regulatory B,
MPC2	H/E	H: CMP, MEP<br>E: Erythrocyte
MS4A3	H/E	H: CMP, GMP, LMPP, MEP<br>E: Erythrocyte
PIM1	E	Erythrocyte
RGS1	B	Memory B, Plasma
S100A4	H/E/M	H: CMP, MEP<br>E: Erythrocyte<br>M: Intermediate monocyte, Non-classical monocyte
SEC63	H	GMP, LMPP
TMEM14B	E	Erythrocyte
TYMS	H/B/E/M	H: CMP, GMP, LMPP<br>B: Pre-B, Regulatory B<br>E: Erythrocyte<br>M: cMOP, hMDP, Pre-monocyte

References

[1] Bingsheng, He, Hai, Yang and Shengli, Wang (2000). Alternating direction method with self-adaptive penalty parameters for monotone variational inequalities. Journal of Optimization Theory and Applications, 106(2), 337-356.

[2] Pratapa, A. et al. (2020).Benchmarking algorithms for gene regulatory network inference from single-cell transcriptomic data. Nature methods, 17(5), 147-154.

[3] Shengli, Wang and Lizhi, Liao (2001). Decomposition method with a variable parameter for a class of monotone variational inequality problems. Journal of Optimization Theory and Applications, 109(2), 415-429.

[4] Street, K. et al. (2018). Slingshot: Cell lineage and pseudotime inference for single-cell tran- scriptomics. BMC Genomics, 19, 447.

[5] Home, P. et al. (2017) Genetic redundancy of GATA factors in the extraembryonic trophoblast lineage ensures the progression of preimplantation and postimplantation mammalian development. Development 144(5): 876-888.

[6] Artus, J., Panthier, J.J. and Hadjantonakis, A.K. (2010) A role for PDGF signaling in expansion of the extra-embryonic endoderm lineage of the mouse blastocyst. Development 137(20): 3361-3372.

[7] Niakan, et al. (2010) Sox17 promotes differentiation in mouse embryonic stem cells by directly regulating extraembryonic gene expression and indirectly antagonizing self-renewal. Genes $\wedge$ Development 24(3):312-26.

[8] McDonald, A. et al. (2014) Sox17-mediated XEN cell conversion identifies dynamic networks controlling cell-fate decisions in embryo-derived stem cells. Cell Reports 9(2):780-93.

[9] L. Bai, G. Shi, X. Zhang, and et al. Transgenic expression of BRCA1 disturbs hematopoietic stem and progenitor cells quiescence and function. *Exp Cell Res.*, 319(17):2739–46, 2013.

[10] A. D. Desgardin, T. Abramova, T. O. Rosanwo, S. Kartha, E.-H. Shim, S. M. Jane, and J. M. Cunningham. Regulation of delta-aminolevulinic acid dehydratase by kruppel-like factor 1. *PLOS ONE*, 7(10):1–11, 2012.

[11] M. Flori, C. A. Schmid, E. T. Sumrall, and et al. The hematopoietic oncoprotein FOXP1 promotes tumor cell survival in diffuse large B-cell lymphoma by repressing S1PR2 signaling. *Blood*, 127(11):1438–1448, 2016.

[12] F. E. Hout, J. van Duren, D. Monteferrario, E. Brinkhuis, N. Mariani, T. M. Westers, D. Chitu, G. Nikoloski, A. A. van de Loosdrecht, B. A. van der Reijden, J. H. Jansen, and G. Huls. Tcf4 promotes erythroid development. *Experimental Hematology*, 69:17–21, 2019.

[13] T. Hu, S. Ghazaryan, C. Sy, and et al. Concomitant inactivation of Rb and E2f8 in hematopoietic stem cells synergizes to induce severe anemia. *Blood*, 119(19):4532–4542, 05 2012.

[14] B. J. Laidlaw and J. G. Cyster. Transcriptional regulation of memory b cell differentiation. *Nature Reviews Immunology*, 21:209–220, 2021.

[15] R. Lakhan and C. V Rathinam. Deficiency of Rbpj Leads to Defective Stress-Induced Hematopoietic Stem Cell Functions and Hif Mediated Activation of Non-canonical Notch Signaling Pathways. *Front Cell Dev Biol.*, 8:622190, 2021.

[16] F. X. Li, J. W. Zhu, C. J. Hogan, and et al. Transgenic expression of BRCA1 disturbs hematopoietic stem and progenitor cells quiescence and function. *Mol Cell Biol.*, 23(10):3607–22, 2003.

[17] S. Mallampati, B. Sun, Y. Lu, H. Ma, Y. Gong, D. Wang, J.-S. Lee, K. Lin, and X. Sun. Integrated genetic approaches identify the molecular mechanisms of Sox4 in early B-cell development: intricate roles for RAG1/2 and CK1ϵ. *Blood*, 123(26):4064–4076, 2014.

[18] J. B. Menendez-Gonzalez, M. Vukovic, A. Abdelfattah, and et al. Gata2 as a Crucial Regulator of Stem Cells in Adult Hematopoiesis and Acute Myeloid Leukemia. *Stem Cell Reports.*, 13(2):291–306, 2019.

[19] I. M. Min, G. Pietramaggiori, F. Kim, E. Passegué, K. Stevenson, and A. J. Wagers. The transcription factor EGR1 controls both the proliferation and localization of hematopoietic stem cells. *Cell Stem Cell*, 2(4):380–391, 2008.

[20] A. R. Moreno. Role of Notch/RBPjk signaling pathaway in embryonic hematopoiesis. *PhD Thesis*, 2007.

[21] S. Muthukkumar, S. Han, V. Rangnekar, and S. Bondada. Role of Egr-1 gene expression in B cell receptor-induced apoptosis in an immature B cell lymphoma. *J Biol Chem.*, 272(44):27987–27993, 1997.

[22] C. Naudin, A. Hattabi, F. Michelet, Miri-Nezhad, and et al. PUMILIO/FOXP1 signaling drives expansion of hematopoietic stem/progenitor and leukemia cells. *Blood*, 129(18):2493–2506, 2017.

[23] L. Pevny, C. Lin, V. D'Agati, M. Simon, S. Orkin, and F. Costantini. Development of hematopoietic cells lacking transcription factor GATA-1. *Development*, 121(1):163–172, 1995.

[24] V. Ramos-Mejía, O. Navarro-Montero, V. Ayllón, and et al. HOXA9 promotes hematopoietic commitment of human embryonic stem cells. *Blood*, 124(20):3065–3075, 11 2014.

[25] E. B. Rankin and K. M. Sakamoto. *The Cellular and Molecular Mechanisms of Hematopoiesis*, pages 1–23. Springer International Publishing, Cham, 2018.

[26] M. A. Rivas, C. Meydan, C. R. Chin, M. F. Challman, and et al. Smc3 dosage regulates B cell transit through germinal centers and restricts their malignant transformation. *Nat Immunol.*, 22(2):240–253, 2021.

[27] B. Sun, S. Mallampati, Y. Gong, D. Wang, V. Lefebvre, and X. Sun. Sox4 is required for the survival of pro-b cells. *The Journal of Immunology*, 190(5):2080–2089, 2013.

[28] A. Vasanthakumar, S. Arnovitz, R. Marquez, and et al. Brca1 deficiency causes bone marrow failure and spontaneous hematologic malignancies in mice. *Blood*, 127(3):310–313, 01 2016.

[29] T. Wang and J. S. Welch. Smc3 Haploinsufficiency and Smc3 Deletion Alter Hematopoiesis In Vivo. *Blood*, 128(22):2903–2903, 2016.

[30] C.-T. Yang, R. Ma, R. A. Axton, M. Jackson, A. H. Taylor, A. Fidanza, L. Marenah, J. Frayne, J. C. Mountford, and L. M. Forrester. Activation of KLF1 Enhances the Differentiation and Maturation of Red Blood Cells from Human Pluripotent Stem Cells. *Stem Cells*, 35(4):886–897, 2017.

[31] T. Yokota, T. Sudo, I. Tomohiko, D. Yukiko, I. Michiko, O. Kenji, and K. Yuzuru. Complementary regulation of early b-lymphoid differentiation by genetic and epigenetic mechanisms. *Int J Hematol*, 98:382–389, 2013.
